# MRPrimer: a MapReduce-based method for the thorough design of valid and ranked primers for PCR

**DOI:** 10.1093/nar/gkv632

**Published:** 2015-06-24

**Authors:** Hyerin Kim, NaNa Kang, Kang-Wook Chon, Seonho Kim, NaHye Lee, JaeHyung Koo, Min-Soo Kim

**Affiliations:** 1 Department of Information and Communication Engineering, DGIST, 333, Techno Jungang Daero, Daegu, 711-873, South Korea; 2 Department of Brain and Cognitive Sciences, DGIST, 333, Techno Jungang Daero, Daegu, 711-873, South Korea

## Abstract

Primer design is a fundamental technique that is widely used for polymerase chain reaction (PCR). Although many methods have been proposed for primer design, they require a great deal of manual effort to generate feasible and valid primers, including homology tests on off-target sequences using BLAST-like tools. That approach is inconvenient for many target sequences of quantitative PCR (qPCR) due to considering the same stringent and allele-invariant constraints. To address this issue, we propose an entirely new method called MRPrimer that can design all feasible and valid primer pairs existing in a DNA database at once, while simultaneously checking a multitude of filtering constraints and validating primer specificity. Furthermore, MRPrimer suggests the best primer pair for each target sequence, based on a ranking method. Through qPCR analysis using 343 primer pairs and the corresponding sequencing and comparative analyses, we showed that the primer pairs designed by MRPrimer are very stable and effective for qPCR. In addition, MRPrimer is computationally efficient and scalable and therefore useful for quickly constructing an entire collection of feasible and valid primers for frequently updated databases like RefSeq. Furthermore, we suggest that MRPrimer can be utilized conveniently for experiments requiring primer design, especially real-time qPCR.

## INTRODUCTION

A primer is a short, single-stranded DNA molecule that serves as a starting point for DNA synthesis. DNA primers are widely used in many biological and medical laboratory techniques that involve DNA polymerase, such as DNA sequencing and polymerase chain reaction (PCR). As a standard laboratory technique for fast mass duplication of specific DNA sequences, PCR with suitable primers is used in a wide variety of applications, including phylogenetic analysis of DNA from different species to detect and identify unknown and distantly related gene sequences (1–3), genetic testing of DNA samples to detect the presence of disease-associated genetic mutations (4), the study of infectious diseases such as HIV and antibiotic-resistant microorganisms (4), PCR-based genetic fingerprinting and parental testing in forensics (4) and microsatellite detection using molecular markers in population biology (5). In addition, quantitative PCR (qPCR), also known as real-time PCR, has been widely used to confirm the results of high-throughput experiments by validating expression changes of selected genes (6). The success of PCR-based experiments, including qPCR analysis, depends strongly on the design of suitable primers against the target sequence(s).

When designing primers, we must simultaneously consider many kinds of constraints, including primer length, melting temperature, GC content, self-complementarity, continuous residues, free-energy value, differences in length and melting temperature between members of primer pairs, product size and pair-complementarity (7). Manual design of primers is time-consuming and may easily yield incorrect results; therefore, automatic methods that can check the aforementioned *single* and *pair* filtering constraints are preferred (8). Additional important constraints that should be evaluated are *homology tests*, i.e. whether the designed primers can only amplify the target sequence(s) rather than off-target sequences; such tests usually require an additional BLAST-like tool. Furthermore, if we want to design a large number of primers for qPCR in a short time that satisfy the same set of filtering constraints (e.g. similar product sizes), the problem becomes much more difficult. Consequently, designing primers is still a difficult problem that has not yet been solved.

The existing methods for primer design can be categorized into two groups, depending on whether we wish to specify a single target sequence or multiple target sequences. The major methods in the former group include Primer3Plus (9) and PrimerBlast (10). Primer3Plus allows users to specify a series of filtering constraints that the primers must satisfy; however, it does not perform homology tests on off-target sequences and therefore requires the user to perform time-consuming tests with a BLAST-like tool for each candidate primer pair. Unlike Primer3Plus, PrimerBlast performs homology tests; however, it specifies only a few filtering constraints, which makes it difficult to design primers as precisely as desired. The major methods in the latter group include CODEHOP (1,11), iCODEHOP (12), GeneFISHER/GeneFISHER2 (13,14), HYDEN (15), FAS-DPD (16), DePiCt (17), Amplicon (18) and SCPrimer (19). All of these methods design degenerate primers, which are actually mixtures of similar, but not identical primers. Most of them design primers by first aligning multiple target sequences to find conserved regions and then designing primer pairs over those conserved regions. For example, CODEHOP and iCODEHOP align target sequences with CLUSTALW (20) and design hybrid degenerate primers that contain a short 3′ degenerate core region of about ∼11–12 bp and a longer 5′ consensus clamp region of ∼18–25 bp. SCPrimer builds phylogenetic trees from aligned multiple sequences to identify candidate primers and then performs a set-covering algorithm to determine the minimal set of primers required to amplify all members of the alignment. Some tools, such as HYDEN and DePiCt, do not rely on multiple sequence alignment (MSA) for primer design, but still rely on heuristic techniques such as greedy hill climbing.

All the previous methods mentioned above have the following three fundamental problems or drawbacks. First, the existing methods for a single target sequence do not support both specification of abundant filtering constraints and homology testing on off-target sequences. In terms of computation, it is a non-trivial problem to support both in a combined manner because this approach typically requires complex and large-scale join computation between a large number of candidate primer pairs designed from each target sequence, as well as a huge number of off-target sequences. Accordingly, users usually use a tool chain of both approaches with some human intervention, but such an approach cannot yield complete results. Second, the existing methods for a single target sequence only focus on designing primers for a specific target sequence; therefore, they cannot be easily used for qPCR, which requires a large number of primer pairs to satisfy the same stringent and allele-invariant constraints (e.g. very similar product sizes) across target sequences. To alleviate this issue, PrimerBank (6,7) was built and updated over the past several years; this database contains 248 578 primer pairs designed from 17 076 human and 18 086 mouse genes following similar constraints. Third, existing methods cannot find all possible primers completely, especially for multiple target sequences. This deficiency is mainly due to the first step, i.e. MSA. The complexity of optimal MSA is inherently NP-complete (21) and so finding an optimal alignment is computationally infeasible for more than a few sequences. Most tools for MSA (e.g. CLUSTALW) (20) are heuristic; therefore, primers designed based on MSA results are also incomplete. Moreover, although we could compute the optimal MSA for a given set of sequences, it would be hard to find all possible primers only with a single fixed alignment because some primers might exist in conserved regions of non-optimal alignments. Methods not based on MSA, like HYDEN, are also heuristic and therefore cannot find all primers. HYDEN also has the serious drawback that it cannot change primer constraints freely (22). Overall, the existing methods tend to miss a large proportion of the feasible primers for given target sequences, even when such primers actually exist.

**Figure 1.**
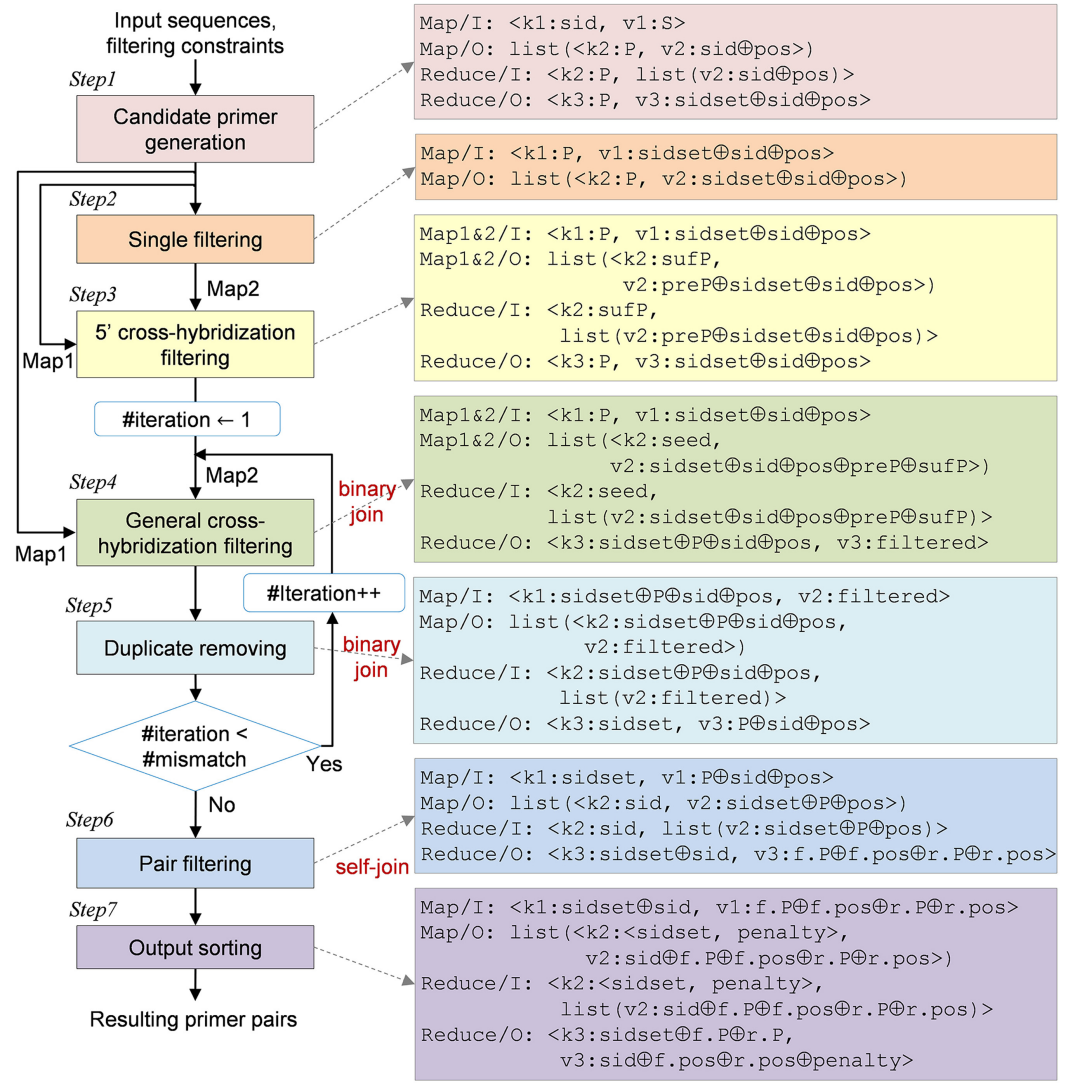
Overall flow of the seven-step MRPrimer method. The boxes on the right show the input/output formats of Map and Reduce for each step. Map/I indicates the input format of Map, Map/O indicates the output format of Map, Reduce/I indicates the input format of Reduce and Reduce/O indicates the output format of Reduce. Map1&2/I indicates the input formats of Map1 and Map2, and Map1&2/O indicates the output formats of Map1 and Map2, in Steps 3 and 4.

In this paper, we propose an entirely new method called *MRPrimer* (http://MRPrimer.com) that overcomes most of the drawbacks of existing methods. For a given set of filtering constraints and a given sequence database (e.g. human gene DNA sequences), the proposed method designs all feasible primers that satisfy the constraints while validating their specificity in one sitting. It finds not only all primers that can amplify a specific single target sequence, but also all primers that can amplify specific multiple target sequences. It neither relies on MSA nor heuristics; instead, it simply finds every possible non-degenerate primer, without missing any feasible or valid primer in the given sequence database, in a single execution. Consequently, it can design a tremendous number of feasible and valid primer pairs, e.g. about 63 million pairs from human genes and 84 million pairs from mouse genes in the consensus coding sequence (CCDS) database (http://www.ncbi.nlm.nih.gov/CCDS/CcdsBrowse.cgi) and show very high coverage ratios, 95% for human and 96% for mouse, for the same database. For realizing the above desirable features, MRPrimer follows a fairly complicated but parsimonious flow of computation based on the MapReduce framework (23). A brief summary of MapReduce is described in the Supplementary Data available at NAR online (Supplementary Figure S1). Figure 1 shows the overall flow of MRPrimer, which is composed of a total of seven steps. Here, each step is a carefully designed MapReduce algorithm.

In addition to designing all feasible and valid primer pairs, while simultaneously checking a multitude of filtering constraints and validating primer specificity, MRPrimer suggests the best primer pair for each target sequence, based on a ranking method performed in its seventh step (i.e. the final step). Consequently, users only need to use the best primer pair(s) for target sequence(s) for their experiments. In addition, the flow of MRPrimer is highly efficient and scalable in terms of computation and so can construct a collection of all primer pairs corresponding to genome-scale data within a few hours using only a small cluster of computers. Therefore, MRPrimer is useful for quickly constructing an entire collection of feasible and valid primers for frequently updated databases like RefSeq. We explained the MRPrimer method in more detail and showed its results in biological experiments. Although MRPrimer can design primers for multiple target sequences, in this paper we focus on qPCR experiments using primers for single target sequences. Especially, we demonstrated the results of qPCR analysis using 343 primer pairs and the corresponding sequencing and comparative analyses for validating the stability and effectiveness of MRPrimer for qPCR.

## MATERIALS AND METHODS

### MRPrimer method

The flow of MRPrimer consists of seven steps (Figure 1). MRPrimer takes a DNA sequence database and a set of filtering constraints as input, and then, over seven steps, it returns all feasible and valid primer pairs that exist in the database. Below, we explain each step in detail.

Step 1 (*candidate primer generation*): Step 1 takes a set of DNA sequences and extracts all possible subsequences of the lengths between the minimum length and the maximum length from each sequence, as candidate primers. Those lengths are specified by users as one of the single-primer filtering constraints. This step also extracts their reverse complementary primers while tagging them with ‘reverse primers’.

Step 2 (*single filtering*): Step 2 applies six filtering constraints for a single primer to each candidate primer passed from Step 1. The primers that violate any filtering constraint are filtered out. The constraints include melting temperature, GC content, self-complementarity, 3′-end self-complementarity, contiguous residue and Gibbs free energy. The primer length was already checked in Step 1. All of these constraints can be specified by users when starting the program. For calculating the melting temperature and the value of free energy, we adopted the nearest-neighbor thermodynamic model (24), which is an improved model of the previous one (25) used in Primer3Plus (9).

Step 3 (*5′ cross-hybridization filtering*): Step 3 eliminates a candidate primer that is the same as any subsequence of an off-target sequence at the 3′ end and has only few mismatches (up to four mismatches) at the 5′ end, and so, might cross-hybridize with the off-target sequence due to the high similarity between them, especially at the 3′ end. This step takes two inputs, *Map1*, which is the output of Step 1 (i.e. all possible subsequences), and *Map2*, which is the output of Step 2 (i.e. a set of candidate primers that passed all single filtering constraints). While performing an all-pair join between the two sets, if a primer from Map1 and a primer from Map2 are identical with each other except at the 5′ end, the primer from Map2 is filtered out. We present an example of this step in Supplementary Figure S2.

Step 4 (*general cross-hybridization filtering*): Step 4 eliminates a candidate primer that is similar with any subsequence of an off-target sequence. This step takes two inputs, *Map1*, which is the output of Step 1 (i.e. all possible subsequences) and *Map2*, which is the output of Step 3 (i.e. a set of candidate primers that passed both the single filtering constraints and 5′ cross-hybridization filtering constraint). We denote the number of mismatched residues as *#mismatch*. While performing an all-pair join between two sets, if a primer from *Map1* and a primer from *Map2* are identical except for #mismatch, the primer from *Map2* is filtered out. To compute this efficiently, this step splits each primer into a set of smaller pieces (called seeds). According to the theorem, a sequence of length *m* with at most *k* mismatches must contain a seed exactly matched of at least ⌊*m*/(*k* + 1)⌋ residues (26–28). All primers from *Map1* and *Map2* having a common seed are collected through the shuffle process of MapReduce (Supplementary Figure S1). Then, this step checks the general cross-hybridization filtering constraint on each group of primers having a common seed. We present an example of this step in Supplementary Figure S3.

Step 5 (*duplicate removing*): After Step 4, there still might be false-positive primers violating the general cross-hybridization filtering constraint. For instance, in Supplementary Figure S3, primer (d) passes Step 4 when it is checked against primer (a). However, it should be filtered out because it is very similar to the primer (b). In order to eliminate such a primer, this step checks every seed of a primer. For instance, among three seeds of primer (d) at the iteration of #mismatch = 2, the common seed between primers (d) and (a) does not violate the general cross-hybridization filtering constraint, whereas the common seed between primers (d) and (b) violates it. Thus, Step 5 eliminates primer (d). The series of Steps 4 and 5 is performed repeatedly until checking of the general cross-hybridization filtering constraint is finished.

Step 6 (*pair filtering*): Step 6 rearranges the result of Step 5 to a set of groups of primers, where each group consists of the primers extracted from the same DNA sequence, by using the shuffle process of MapReduce (Supplementary Figure S1). Then, this step splits the candidate primers of each group into two sets, a set of forward primers and a set of reverse primers, using tags addressed in Step 1 and performs self-join computation between them. In self-join computation, this step applies five filtering constraints to each candidate primer pair. These constraints include length difference, melting temperature difference, product size, pair-complementarity and 3′-end pair-complementarity. They all can be specified by users when starting the program.

Step 7 (*ranking*): The primer pairs passed from Step 6 might not be equally effective even if they satisfy all the given constraints. Thus, the final step of MRPrimer, i.e. Step 7, determines their ranking by calculating a penalty score for each primer pair. The ranking is determined within a specific target sequence(s) and so users can easily pick the top-1 primer pair for each target sequence. The calculation of penalty scores follows the method of Primer3Plus (9), which adds penalty scores of seven constraints for single primers and five constraints for primer pairs. In general, for the constraints having a range (e.g. melting temperature), the median value has the lowest penalty. For the other constraints (e.g. self-complementarity), the smallest value, typically zero, has the lowest penalty. Each penalty score for each constraint is normalized between 0 and 1. Primer pairs with low scores have high rank for the corresponding target sequence.

### Access to MRPrimer

The most recent MRPrimer release is available at http://MRPrimer.com. The source code for MRPrimer is freely available under the GNU General Public License (GPL) version 3.0.

### Validating the completeness and ranking method of MRPrimer

To show the completeness and superiority of MRPrimer in terms of the number of primer pairs designed, we compare the results of MRPrimer with PrimerBank, which is one of the largest databases of primers that has been built and updated over the past several years (6,7,29,30). PrimerBank uses the human and mouse genes databases of the NCBI RefSeq project (31–33). There are multiple versions of the RefSeq database, specified by their release dates. Unfortunately, the version of the RefSeq database used for PrimerBank is out of date and is therefore no longer available. Thus, we use the oldest version available for comparison because it is the version most similar to that used for PrimerBank. That version (released on 07 November 2007) contains a total of 22 942 human mRNA sequences and a total of 27 305 mouse mRNA sequences. For a fair comparison, we use the exact same set of filtering constraints as were used to construct PrimerBank (6); these constraints are summarized in Supplementary Table S1.

In addition, to show the effectiveness of ranking method of MRPrimer, we extracted the validated primer pairs that specifically cover mouse olfactory receptor (OR) sequences from PrimerBank and analyzed them using the ranking method of MRPrimer. Among 27 305 mouse mRNA sequences of the RefSeq database, there are 990 mouse OR genes. We searched for the NCBI Gene IDs of those genes in the PrimerBank (http://pga.mgh.harvard.edu/primerbank/index.html) and collected 778 validated primer pairs covering 768 mouse OR genes. MRPrimer can also find 772 out of 778 primer pairs and so we can rank those 772 primer pairs, which are common between PrimerBank and MRPrimer, according to the ranking method of MRPrimer. The ranking results revealed the rationality of our ranking method. Six primer pairs were not found by MRPrimer because the six sequences containing them are not present in the version of the RefSeq database (released on 07 November 2007) used for our experiments.

### qPCR analysis of MRPrimer

To validate the quality of primer pairs designed by MRPrimer, we performed qPCR experiments using the mouse CCDS database rather than the RefSeq database (http://www.ncbi.nlm.nih.gov/CCDS/CcdsBrowse.cgi). It provides a gold standard for coding-region locations (34,35). In the CCDS datasets, there are currently a total of 29 064 human gene DNA sequences (the last update was 29 November 2013) and a total of 23 874 mouse gene DNA sequences (the last update was 07 April 2014). We primarily used mouse genes for our qPCR analysis.

We randomly selected 96 OR genes and 99 non-OR genes (including pheromone receptors, G proteins, ion channels, signaling molecules, etc.). Thus, we performed a total of 195 qPCR experiments. For each gene, we selected the top-1 primer pair for that gene, according to the ranking method of MRPrimer. We summarize the forward and backward primers designed and selected automatically by MRPrimer in Supplementary Tables S2 and S3. We followed the MIQE guidelines (36) for the qPCR experiments.

### Comparative analysis between MRPrimer and PrimerBank

To demonstrate the effectiveness and superiority of MRPrimer for qPCR, we performed both qPCR and sequencing analyses and compared the results with those obtained using PrimerBank. Because the primers of both MRPrimer and PrimerBank easily succeeded in amplifying normal target sequences, we compared their performance using ‘difficult’ target sequences, i.e. OR genes that have many homologous regions and, therefore, often fail in qPCR experiments (30). OR genes form the largest multigene family in mammals (37). These genes share many homologous regions; consequently, it is difficult to design valid primer pairs for them (30). A number of studies reported the expression of ORs in olfactory as well as non-olfactory tissues (38,39). For such studies, qPCR using valid primer pairs is an effective and simple way to detect OR genes (38,39).

To prepare the mouse OR genes, we searched for the NCBI Gene IDs of the mouse OR genes from CCDS database in PrimerBank and collected 860 validated primer pairs, each of which amplifies a single OR gene. We first checked their specificity using PrimerBlast. Among the 860 primer pairs, 599 primer pairs were of high quality (i.e. high specificity). These 599 primer pairs were 100% matches to their intended expected target genes and the possibility of matching an off-target gene was no more than 80%. Among the remaining 261 primer pairs, 96 were 100% matched with the expected target genes and the possibility of matching an off-target gene was no more than 85%. These 695 (599 plus 96) primer pairs are considered highly specific for their target genes. Among the remaining primer pairs, 75 primer pairs were 95% matched, and 69 primer pairs were 90% matched to both target and off-target genes. These 144 (75 plus 69) primer pairs can amplify target genes along with off-target genes (i.e. wrong target or multi-target). We selected about 6% of the pairs corresponding to the 695 highly specific genes (i.e. 40 primer pairs) and about 24% of the pairs corresponding to the 144 less specific genes (i.e. 34 primer pairs). Next, we selected 74 primer pairs from the results of MRPrimer for the same 74 genes. The selection ratios differed (6 versus 24%) because the 695 genes are relatively easy to amplify, whereas the 144 genes are relatively hard. We also note that the 74 OR genes used for this experiment are distinct from the 96 OR genes in the above experiment; furthermore, they represent harder target sequences. We summarize the forward and backward primers for the 74 genes of MRPrimer and PrimerBank used in our experiments (Supplementary Table S4).

To identify amplified samples, we compared the sequences of qPCR amplicons with the expected gene sequences using NCBI BLASTn (http://blast.ncbi.nlm.nih.gov/Blast.cgi) and checked the percent identity between the two sequences. For BLAST analysis, we applied the following criteria, used in a previous study (30). If more than 50% of the length of an expected PCR product sequence matches with only the expected target sequence, multiple genes or another gene with 100% identity between the sequences, it is considered to be *target-specific*, *multiple-target* or *wrong target*, respectively. Finally, if a qPCR product sequence does not match with at least 50% of the length of its expected target sequence, it is considered to be a *sequencing failure*.

## RESULTS

### The completeness and effective ranking system of MRPrimer

In terms of the number of primer pairs designed, MRPrimer found a much larger number of feasible and valid primer pairs than PrimerBank under the same filtering constraints (Supplementary Table S1). Table 1 shows the number of primer pairs designed by MRPrimer and the number of genes covered by those primer pairs, relative to the corresponding values for PrimerBank. In Table 1, we show that PrimerBank yields a coverage ratio of 94% for their RefSeq database, which is not available now. In terms of the version of the RefSeq database released on 07 November 2007, which is the available version most similar to that used for PrimerBank, the coverage ratio decreases to 78% for human genes and 69% for mouse genes. The size of the RefSeq database is increasing continuously and the latest version (released on 03 February 15) contains a total of 99 722 sequences for human and 128 898 sequences for mouse. However, the number of primer pairs in PrimerBank is fixed, and has not increased since 2012. Because PrimerBank consists of primers collected manually, it is extremely hard to update it according to the release of a new version of the RefSeq database. By contrast, MRPrimer is not a static collection, but a program that can generate a collection immediately when given a new version of a database. For the same RefSeq database, the coverage ratios for MRPrimer (88 and 81%) are much higher than those for PrimerBank (78 and 69%). In addition, for up-to-date human and mouse CCDS databases, MRPrimer exhibits the highest coverage ratios ever: 95% for human and 96% for mouse. These impressive ratios are mainly due to the high quality of the CCDS database.

**Table 1.** The statistics of PrimerBank and the results of MRPrimer

	PrimerBank^a^		PrimerBank^b^		MRPrimer^c^		MRPrimer^d^	
Datasets	Human N/A	Mouse N/A	Human 22 942	Mouse 27 305	Human 22 942	Mouse 27 305	Human 29 064	Mouse 23 889
# of primer pairs	129 692	118 886	129 692	118 886	63 419 755	86 867 667	63 632 594	84 226 391
# of genes covered	17 973	18 955	17 973	18 955	20 199	22 253	27 980	22 798
Coverage ratio	94%		78%	69%	88%	81%	95%	96%

N/A indicates datasets that are not available.

^a^Statistics are from PrimerBank (6).

^b^Statistics are the same as with ^a^, but the dataset is the RefSeq database (released on 07 November 2007), the available data set most similar to the one used for ^a^.

^c^The dataset and filtering constraints (Supplementary Table S1) are the same as in ^b^. Statistics are from MRPrimer.

^d^The dataset is the CCDS database and the filtering constraints are the same as in ^b^. Statistics are from MRPrimer.

**Figure 2.**
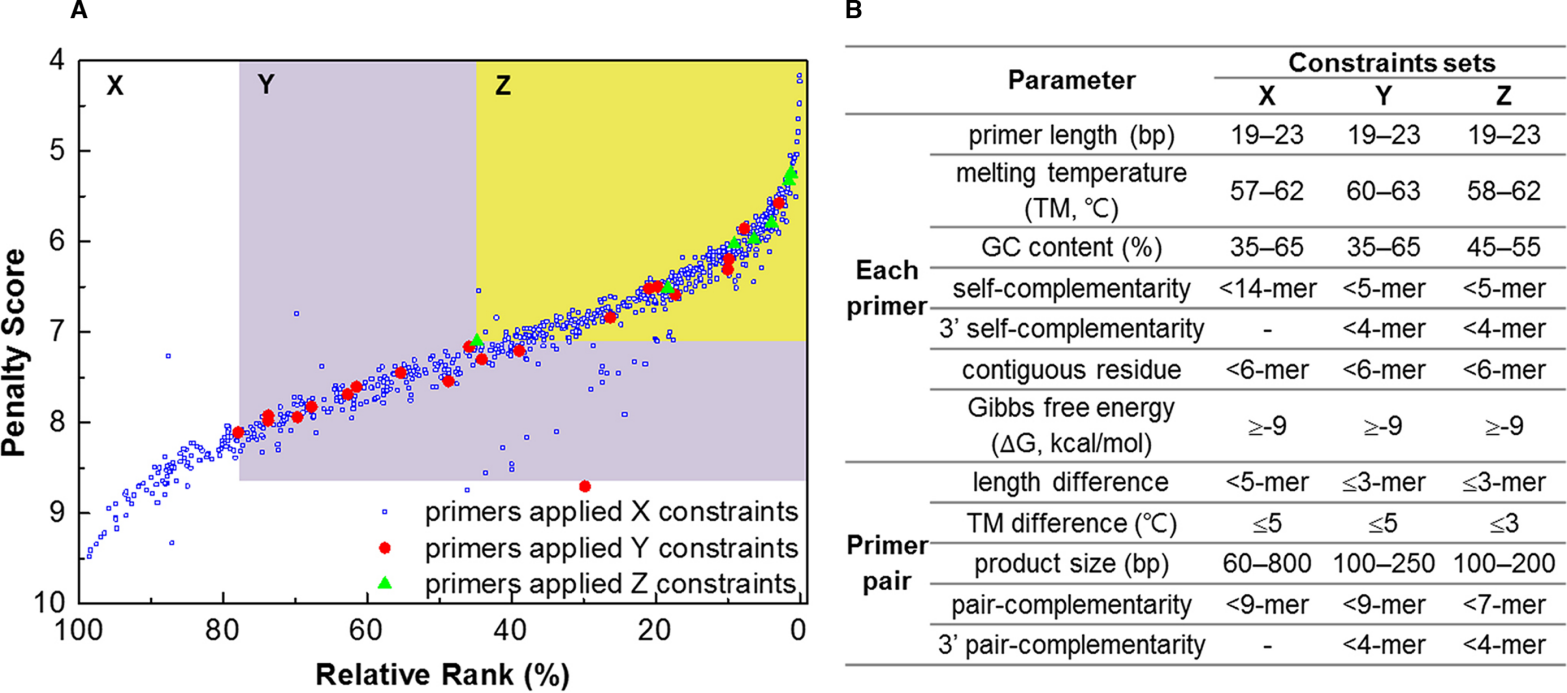
The advantage of the ranking method of MRPrimer. Primer pairs following strict constraints have high ranks and *viceversa*. The top-1 primer pair of MRPrimer indicates that it follows the strictest constraints for the target sequence. (**A**) Distribution plot of 772 primer pairs specifically covering mouse OR genes; these primer pairs are common to PrimerBank and MRPrimer. They are sorted by the ranking method of MRPrimer, in particular by relative rank along the horizontal axis and by penalty scores ranked along the vertical axis. The 737 blue dots are spread over the area of X (i.e. the entire area), the 28 red dots are spread over the area of Y and the 7 green dots are spread over the area of Z (i.e. yellow colored area). (**B**) The sets of filtering constraints corresponding to each area.

MRPrimer yielded effective ranking results for a large number of the resultant primer pairs. We extracted a total of 772 common validated primer pairs that specifically cover mouse OR sequences and analyzed them using the ranking method of MRPrimer. Figure 2A shows the relationship between ranks and penalty scores of those 772 primer pairs. MRPrimer calculates a penalty score for each primer pair and determines the ranking among primer pairs for a specific target sequence, as described in Step 7. Because there are different numbers of primer pairs for each target sequence, we normalized the ranks to between 0 and 100%, denoted as *relative rank* in the figure. Figure 2A shows the strong correlation between ranks and penalty scores. Primer pairs with small penalties have high rank (i.e. small %).

Figure 2B shows three sets of filtering constraints. X is a relatively relaxed constraint, Y is the set of constraints used in PrimerBank and Z is a relatively strict constraint. According to X, Y and Z, the 772 primer pairs can also be categorized into the corresponding three groups designed by using X, Y and Z (denoted as blue, red and green dots, respectively). Although the authors of PrimerBank claimed that they used the Y constraints to construct PrimerBank, we observed that the primer pairs in PrimerBank did not strictly follow the Y constraints, but instead followed the X constraints, which are looser. Groups X, Y and Z contain 737, 28 and 7 primer pairs, respectively. Some primer pairs (blue dots) exist in the area of Y or Z because a primer pair that satisfies all constraints except one (or a few) could have a low penalty score and a high rank. Along with this, we suggest that primer pairs following strict constraints have high ranks and small penalty scores without loss of generality. Because a primer pair with a low penalty score has a high chance of success in amplifying a target sequence (9) and MRPrimer returns the resultant primer pairs ordered by rank, users simply need to select the top-1 primer pair, i.e. the probably best primer pair.

### MRPrimer validation by qPCR analysis

**Figure 3.**
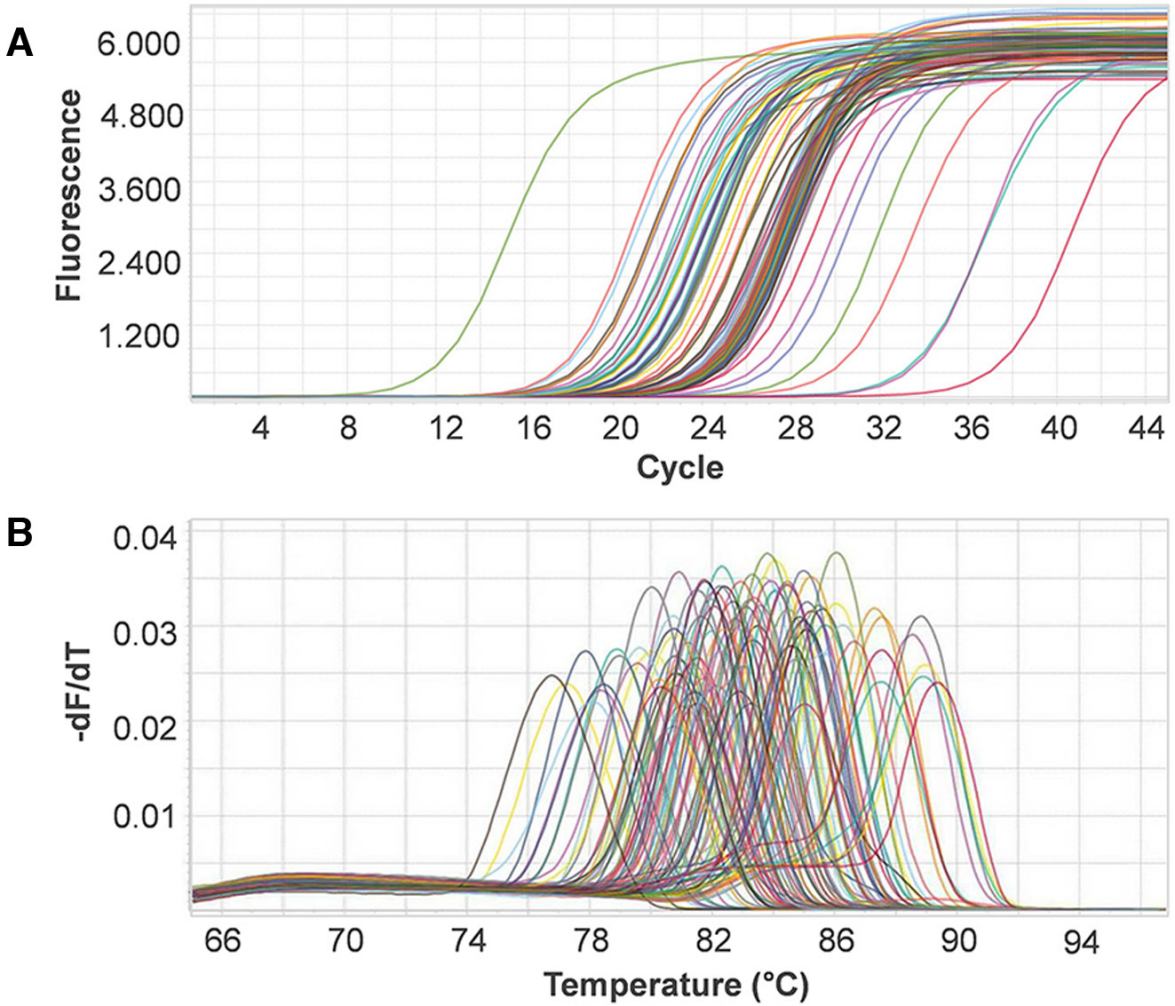
Verification of 99 primer pairs for non-OR genes using qPCR analysis. The target genes were randomly selected from among non-OR genes (including pheromone receptors, G proteins, ion channels, signaling molecules and so on) in the mouse CCDS database. We used the top-1 primer pair automatically designed by MRPrimer. PCR amplification and amplicon dissociation analyses were performed successfully. (**A**) Amplification curves of the target genes. (**B**) Melting curves of the target genes.

**Figure 4.**
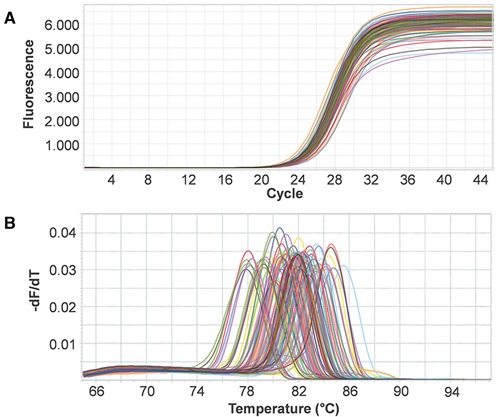
Verification of 96 primer pairs for OR genes using qPCR analysis. The target genes were randomly selected from among OR genes in the mouse CCDS database. As in Figure 3, PCR amplification and amplicon dissociation analyses were performed successfully.

For validation of MRPrimer, we performed qPCR using the top-1 primer pairs designed and selected automatically by MRPrimer, covering 195 genes randomly selected from among the mouse CCDS database (Supplementary Tables S2 and S3). The qPCR results reveal that all primer pairs designed by MRPrimer successfully amplified the corresponding target genes (Figures 3 and 4). Each of the qPCR melting curves clearly yielded a single peak, suggesting that each qPCR product is a single product without off-target gene amplification. We confirmed the qPCR products by sequencing analysis (data not shown), indicating that MRPrimer specifically amplified the corresponding target genes.

### Comparative analysis of MRPrimer and PrimerBank using qPCR and sequencing analyses

**Figure 5.**
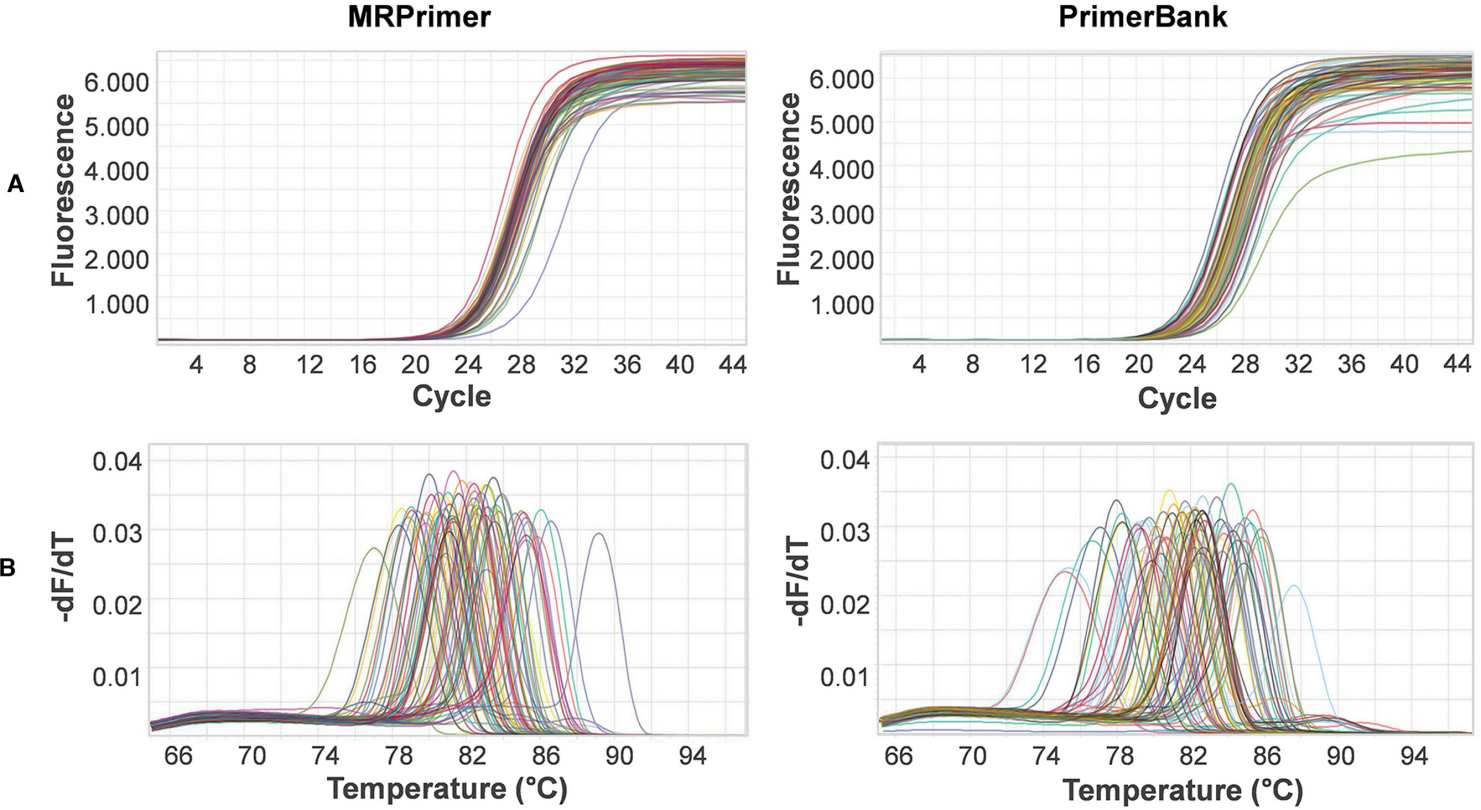
Comparative analysis between MRPrimer and PrimerBank using qPCR analysis. A total of 74 genes were selected from mouse OR genes: 40 genes were relatively easy to amplify (i.e. the primers were highly target-specific), whereas 34 genes were relatively hard (i.e. the primers were highly multi-targets or wrong-targets). The primer pairs for MRPrimer were automatically selected (Supplementary Table S4). Both MRPrimer and PrimerBank primers successfully performed real-time amplification and the final amplicon dissociation process. (**A**) Amplification curves of 74 genes. (**B**) Melting curves of 74 genes. The identities of the amplified products were additionally confirmed by sequencing analysis (Figure 6).

For this comparative analysis, MRPrimer yielded similar results in qPCR analysis and better results in sequencing analysis, relative to PrimerBank. Before starting the experiments, we analyzed primer sets (see ‘Materials and Methods’ section). The selected 74 primer sets (Supplementary Table S4) from MRPrimer and PrimerBank were used to perform qPCR. The result (Figure 5) shows that both MRPrimer and PrimerBank primers successfully amplified even the difficult target sequences like OR genes.

**Figure 6.**
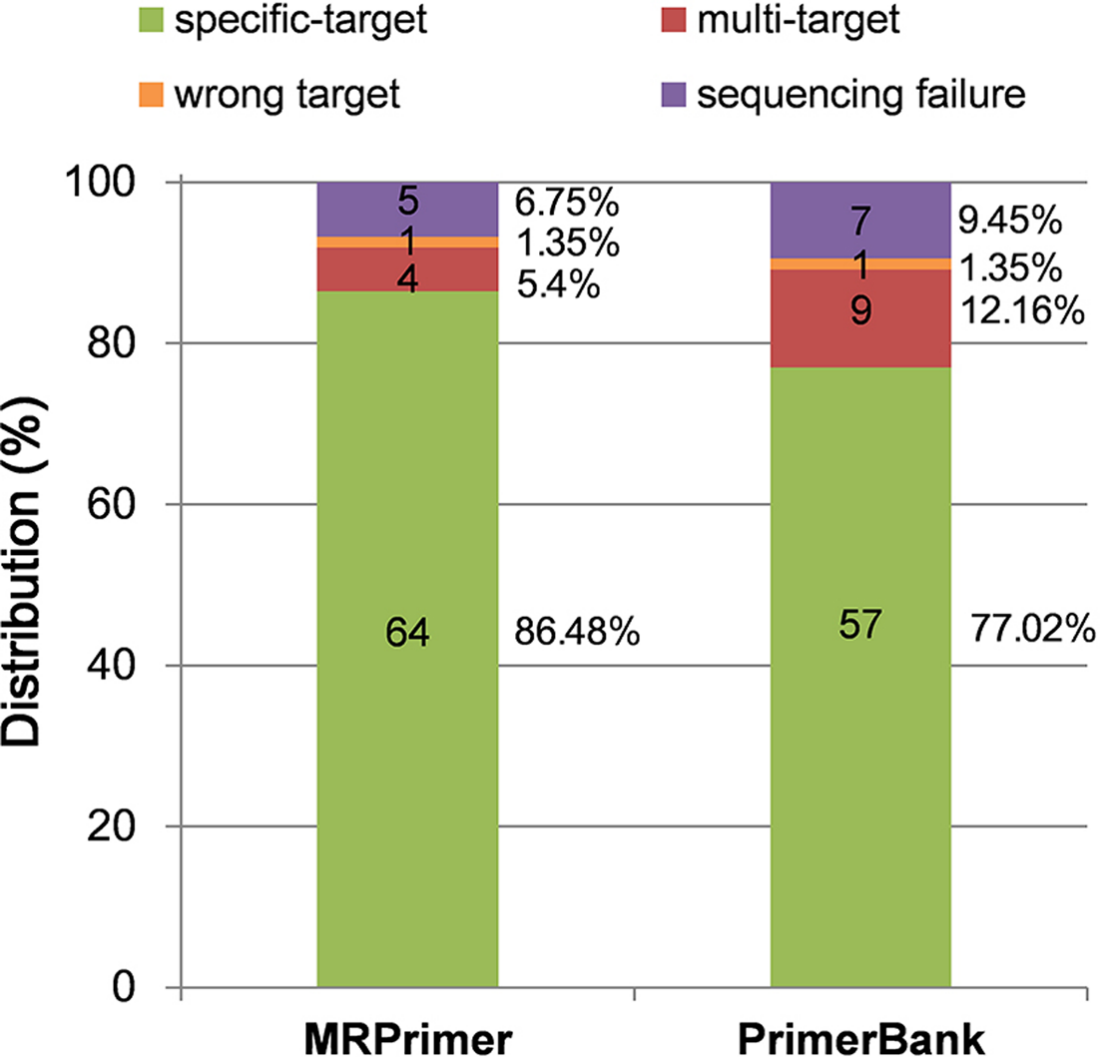
Comparative analysis between MRPrimer and PrimerBank using sequencing analysis. According to the sequencing analysis (see ‘Materials and Methods’ section), in the case of MRPrimer, 64 samples (86.48%) were target-specific, 4 (5.4%) were matched to multiple genes (multi-target) and 1 was matched to another gene (wrong target). The remaining five samples (6.75%) did not satisfy our criteria for sequencing analysis. On the other hand, in the case of PrimerBank, 57 samples (77.02%) were target gene specific, 9 (12.16%) were matched to multiple genes (multi-target) and 1 was matched to another gene (wrong target). The remaining seven samples (9.45%) did not satisfy our criteria for sequencing analysis. These observations suggest that the primers designed by MRPrimer were more effective than the PrimerBank primers.

However, the sequencing analysis yielded somewhat different results. We examined all PCR products by sequencing and compared the qPCR amplicon sequences to the expected gene sequences by NCBI BLASTn. Figure 6 shows the sequencing results. Among the MRPrimer 74 qPCR amplicons, 64 samples (86.48%) were target-specific and these samples were 100% matched to the only expected target. Four samples (5.4%) were matched to both the expected target and an unexpected target at the same time (multi-target). Only one sample was matched to another gene (wrong target). The remaining five samples (6.75%) did not satisfy our criteria for sequencing analysis. On the other hand, among the 74 PrimerBank samples, 57 (77.02%) were target gene specific, 9 (12.16%) were matched to multiple genes (multi-target), one was matched to another gene (wrong target) and seven (9.45%) did not satisfy our criteria for sequencing analysis. Based on these results, we confirmed that a single qPCR peak does not indicate the amplification of a specific single target. Because we intentionally selected difficult target sequences for this comparative analysis, the target-specific ratio of 86.48% does not indicate the overall effectiveness of MRPrimer. These findings suggest that primers designed by MRPrimer were more effective than PrimerBank primers.

## DISCUSSION

Our biological and computational validation results indicate that MRPrimer designs all possible feasible and valid primer pairs for an entire DNA database, and that the resultant primers are very useful and effective for qPCR and sequencing analyses. We can summarize its major advantages in terms of practical usage as follows.

First, MRPrimer performs both single/pair primer filtering and homology tests, in a combined and integrated manner. Furthermore, it automatically sorts the resulting primer pairs for each target sequence, based on penalty scores. Thus, users do not need to be concerned about mistakes when validating a candidate primer. Because it produces a complete set of primer pairs, users can repeatedly reuse the results, unless filtering constraints need to be changed.

Second, MRPrimer designs all feasible primer pairs strictly, following the same filtering constraints. For example, it can design a large number of primers that follow a very strict constraint on product size (e.g. between 100 and 150 bp) for a given set of tens of thousands of sequences all at once. This powerful feature would be especially useful for qPCR experiments.

Third, MRPrimer is computationally efficient and scalable, and able to design entire primer pairs for a whole DNA database within a few hours using only a small-scale cluster of computers. The evaluation of the computational efficiency and scalability of MRPrimer is described in Supplementary Tables S5 and S6. Even for a database of 105 180 DNA sequences, it could design all primer pairs within 7 h (Supplementary Figure S4). This feature is very useful, especially for sequence databases that are updated frequently, like the RefSeq database.

In conclusion, we believe that we have developed an advanced technology that could overcome the drawbacks of existing design methods, while also integrating several desirable features required by researchers in this field into a single method. We also believe that MRPrimer or a variation of MRPrimer could be very useful for other application areas such as DNA construction and genetic engineering. If there is a specific fragment to be amplified from a given DNA template, there are a variety of putative primers that could accomplish this. An MRPrimer-style method of screening and ranking in parallel could be very effective at designing ideal primer pairs for that purpose. For example, it could be effectively used to alleviate the problem of lack of novel primer pairs for detecting unauthorized genetically modified organisms (GMOs) in the collection of GMO detection methods, called GMO Detection method Database (GMDD) (40,41). At this stage, however, MRPrimer is still less than ideal as a primer design method. For example, it does not support a rich web-interface that can search for desired primers instantly while freely varying filtering constraints; these features may be added in future work.

## SUPPLEMENTARY DATA

Supplementary Data are available at NAR Online.

SUPPLEMENTARY DATA
