# Supplementary material for: MRPrimer: a MapReduce-based method for the thorough design of valid and ranked primers for PCR
Source: Nucleic Acids Res. 2015 Jun 24;43(20):e130. doi: 10.1093/nar/gkv632 (PMC4787749; doi:10.1093/nar/gkv632)
Supplement: SUPPLEMENTARY DATA [file supp_43_20_e130__index.html]

MRPrimer: a MapReduce-based method for the thorough design of valid and ranked primers for PCR — MRPrimer: a MapReduce-based method for the thorough design of valid and ranked primers for PCR — SUPPLEMENTARY DATA 

# MRPrimer: a MapReduce-based method for the thorough design of valid and ranked primers for PCR

## SUPPLEMENTARY DATA

- SUPPLEMENTARY DATA
