# Supplementary material for: MRPrimer: a MapReduce-based method for the thorough design of valid and ranked primers for PCR
Source: Nucleic Acids Res. 2015 Jun 24;43(20):e130. doi: 10.1093/nar/gkv632 (PMC4787749; doi:10.1093/nar/gkv632)
Supplement: SUPPLEMENTARY DATA [file supp_gkv632_nar-00857-met-k-2015-File008.pdf]

MapReduce

MRPrimer is based on the MapReduce framework, whose dataflow is shown in Figure S1. One round of MapReduce consists of two user-defined functions, *Map* and *Reduce*. Here, Reduce is optional, and can therefore be omitted. The input data for a round is distributed over the disks of a cluster of computers. The partial data in each computer is processed by Map functions, shuffled via network, processed by Reduce functions, and then stored on the disks; the resultant data is then fed to the Map functions of the next MapReduce round. The input/output format of the data, i.e., the signatures of the Map and Reduce functions, are formally defined as follows.

$$\text{Map: } \langle k1, v1\rangle \rightarrow \text{list}(\langle k2, v2\rangle)$$

$$\text{Reduce: } \langle k2, \text{list}(v2)\rangle \rightarrow \langle k3, v3\rangle$$

The Map function takes a pair of *key* and *value*, ⟨k1, v1⟩, as input (e.g., k1 is a sequence ID, and v1 is the sequence itself), and then returns a list of pairs, ⟨k2, v2⟩, in a different domain as output. The Reduce function takes a pair of key and list of values, i.e., ⟨k2, list(v2)⟩, as input, and then returns a pair of key and value, i.e., ⟨k3, v3⟩, in a different domain as output. Here, we note that v2 in the output of Map becomes list(v2) in the input of Reduce because the shuffle process gathers all v2s having the same k2, which are scattered over computers, into a single list of v2s on a single computer. MRPrimer relies extensively on this feature of MapReduce to efficiently perform large-scale and complicated computation.

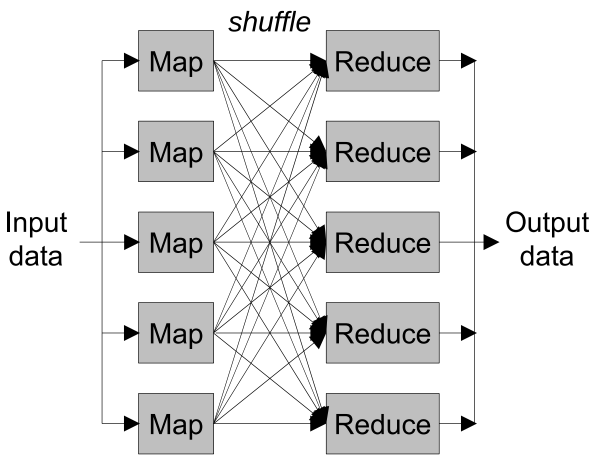

Figure S1. Dataflow of the MapReduce framework.

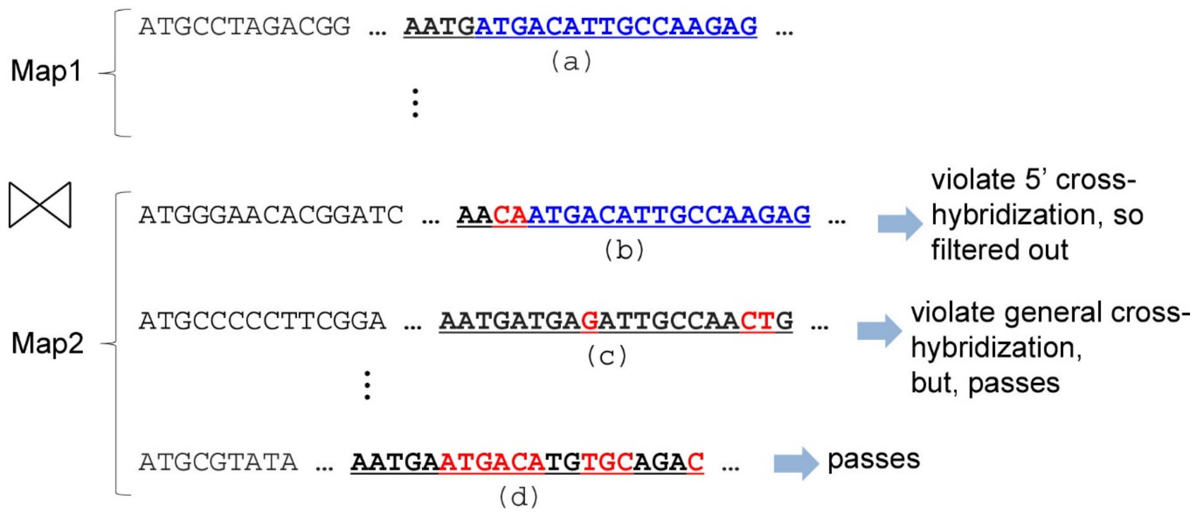

Figure S2. An example of the 5' cross-hybridization filtering step. There is a 20-mer primer (a) in Map1, and three candidate primers of the same length, (b), (c), and (d), in Map2. We assume that the user-defined value for the maximum mismatches at the 5' end is 4. Then, primer (b) is filtered out because it has the same 16-mer subsequence (in blue) as primer (a) in an off-target sequence (only two mismatches in red). Primers (c) and (d) pass because they have no such identical subsequence. However, since it is similar to primer (a), primer (c) might cross-hybridize to the off-target sequence to which primer (a) belongs.

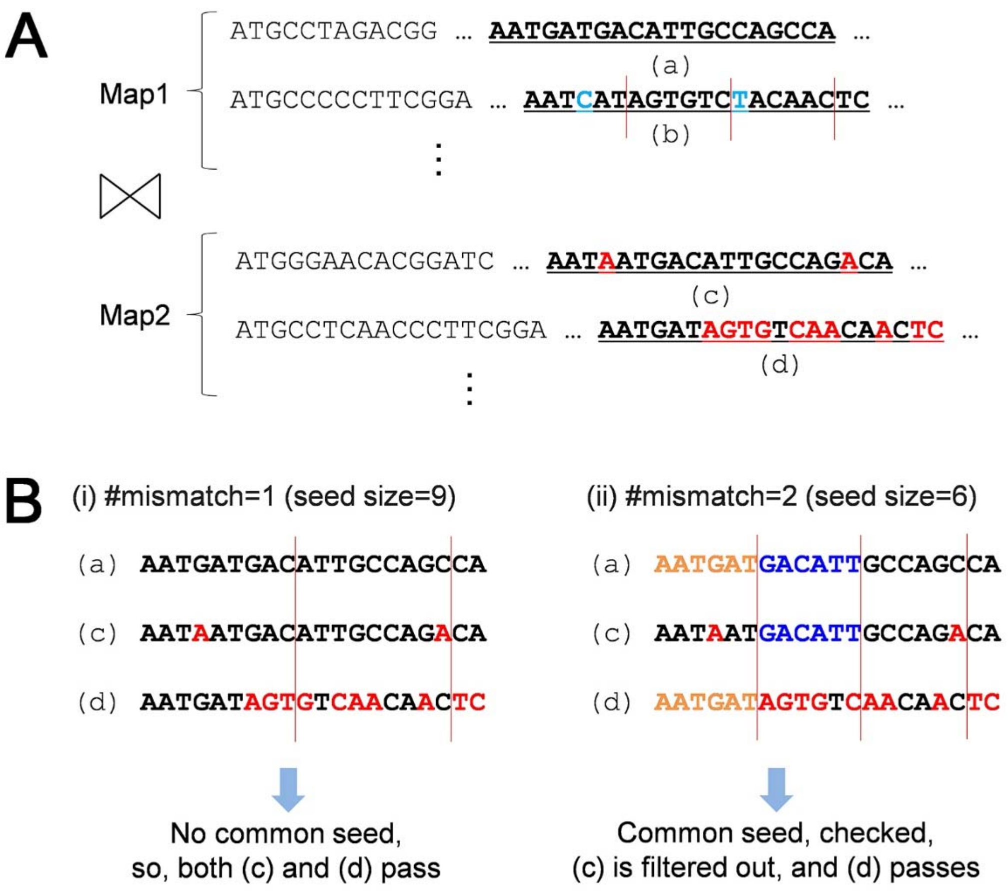

Figure S3. An example of the general cross-hybridization filtering step. (A) There are two primers, (a) and (b), in Map1, and two candidate primers, (c) and (d), in Map2. Primer (c) is similar to primer (a), with only two mismatches (in red). Primer (d) is quite different from primer (a), although it is similar to primer (b), with only two mismatches (in cyan). (B) In the first execution of Steps 4 and 5 with #mismatch = 1, the seed size is nine. Primers (c) and (d) have no common seed with primer (a), and so they pass. In the second execution of Steps 4 and 5 with #mismatch = 2, the seed size is 6. Primer (c) has a common seed (in blue) with primer (a), and so both the prefix and suffix of the seed are checked. Due to the presence of only two mismatches (in red), primer (c) is filtered out. Primer (d) also has a common seed (in orange) with primer (a), and so it is checked. It can pass Step 4 due to the large number of mismatches (in red), but cannot pass Step 5 because it is identified as similar to primer (b).

Table S1. Summary of the filtering constraints used in PrimerBank.

	Parameter	Value
**Each primer**	primer length	19–23 bp
	melting temperature (TM)	60–63℃
	GC content	35–65%
	self-complementarity	<5-mer
	3' self-complementarity	<4-mer
	contiguous residue	<6-mer
	Gibbs free energy (ΔG)	≥-9 kcal/mol
**Primer pair**	length difference	≤3-mer
	TM difference	≤5℃
	product size	100–250 bp
	pair-complementarity	<9-mer
	3' pair-complementarity	<4-mer

Table S2. Primers for non-OR genes used in the biological experiments to validate MRPrimer.

Gene		Sequence	Size (bp)	Gene		Sequence	Size (bp)
Adcy6	F	GACCAACTGCGTAAGGACCAT	178	Gnai2	F	CTTATGACTTGGTGCTGGCTGA	183
	R	TCAGGGTGGAGTATGGGAACA			R	ACTCAGGGAAACAGATGGTCAG	
Adcy9	F	AATGAAACAAGGGGACGAGGAG	119	Gnai3	F	GCAGATGATGCCCGACAGTTA	136
	R	TAAAGGGGCGGAATGCTATCG			R	ATTCCCTGGACCTGCTAAAGC	
Anxa2	F	GAGACGGTGATTTTGGGCCTAT	127	Gnal	F	TGGGACGATGAAGGAGTGAAG	124
	R	GGTTGGTTCGTGAGCAGATGAT			R	GGTCTGTGGGTGTGTAGTCAA	
Anxa4	F	AGAGAAGAGATGGGGGACAGA	168	Gnao1	F	TCACCCTTGACCATCTGCTTTC	107
	R	GCCAACAGGGCATCTTCAAAG			R	TTGTTGGGTGAGCGGTTTTTG	
Cacna1c	F	GCAGCGTAAGGATGAGTGAAGA	170	Gnaq	F	GGTTGATGTGGAGAAGGTGTCT	183
	R	TAGAGAGGCAGAGCGAAGGAA			R	TGTGTAGGCAGATAGGAAGGGT	
Cacna1i	F	AAACGTGCTCCTGCTCTGTTT	159	Gnas	F	TACGATCAGGACGACTACGAGAC	175
	R	TCGTCCTCTTCTGGTTGGTAGT			R	GAGTGAGTGACTGGTTGAAGGT	
Cacnb1	F	AGATGACCGACAACAGGAACC	100	Gnat2	F	TGGACGTCATCAGGAAGTTGT	186
	R	CAGCCCTCCAGCTCATTCTTAT			R	CGATGATGCCTGTTGTCTTGAC	
Clcn3	F	GAGCATCTCGAGCAACTAAAGC	162	Gnaz	F	CAGAGAGCAAGGGTGAGATTACA	139
	R	TTCTGTCTCCTCTCTGTCCTCA			R	AGGTCGTTCAGGTAGTAGGCT	
Clcn4-2	F	TGGAGTCTTTGGGGGTTTATGG	114	Ifnar2	F	ACTGGCCCCTATGAGAGAAGAA	191
	R	ACCGCAATAACCTCCAACACT			R	TCGTCTAGGAGGATGGTGTCTT	
Clcn6	F	ATCCTTGGGGAGACACAGGAA	152	Insig1	F	GCTGTATTGCCGTGTTCGTT	111
	R	CACTTCACCGCCTCGTATCTT			R	TCCACCACAAACCCAAAGAGA	
Clcn7	F	CGACACAGCGTCTAATCACAAC	136	Kcnb1	F	GGAGAAAAATGGAGAGGGCGT	180
	R	GGACCTCTCCACAAACACCTT			R	TTCAAGTGCTGCGGACTAGAC	
Fpr1	F	GGTTCATCATTGGGTTCAGCAC	124	Kcnc3	F	CAGAAGACAAGAGCCCAATCAC	128
	R	ACAAAGGAGAGAACCCGCAAA			R	GCGGGACTTCTCGTAACCTTT	
Fpr2	F	TCCCTGCCTTATAGTCTTGAGAG	108	Kcnc4	F	GGAGGTAGAAACAGAGCCCATT	185
	R	TGGGGCCTTTAACTCAATGTCT			R	ACAAACCACTCAATCCCACCTC	
Fpr3	F	CATTCTCACTTTGCCCCTTTTCC	100	Kcnd1	F	ACTGCAGCCCTGGTTTTCTAC	128
	R	AACAGAGTTGCCCCAGGATAC			R	TCACCACACGACTGCTCTTTG	
Gcg	F	CACCAGCGACTACAGCAAATAC	169	Kcnh2	F	ACCTGCTTACTGCCCTCTACT	133
	R	CTGGCCCTCCAAGTAAGAACT			R	GACTTTCCAGGACGGGCATAT	
Gna11	F	TCCGCACAATCATCACCTACC	136	Kcnh6	F	TACAGCAAATGCCCCCAAGTC	105
	R	CTCTGTGGCCCATCAAACTCA			R	GTCTGTTCATCTGGGCTTGGA	
Gna12	F	CTAGAAAGGCCACCAAGGGAAT	152	Kcnj11	F	TCGTGTCCAAGAAAGGCAACT	110
	R	CGAGGACACCATGAACAGGATA			R	AGTGTGTGGCCATTTGAGGTC	
Gnai1	F	CGGAAGAGGAGTGTAAGCAGTA	152	Kcnj9	F	TCGTCTCACCTCTCGTCATCA	178
	R	CCCAGCAAGCACGAAAAGTT			R	CACAACACTTCATCCACCAGGTA	

Kcnk1	F	TTGTCACCGTTTCCTGCTTCT	157	Syt7	F	GAAAGCCATCAACGACCTAGAC	120
	R	AACTTCTGGTTGTAGCCTTCCC			R	TAAGGGGCGTAGGGTGAAATG	
Kcnk12	F	CTACTTCTGCTTCGTCACCTTCA	159	Syt13	F	AGAACCTCCACTCCAACCAATC	110
	R	ATGGAGATGACGTTGAAGAGCG			R	GGCCCGTTTTGTCTGTTTCTT	
Kcnk16	F	GTCATTCTCATCTTCCCACCCA	117	Taar1	F	GCGGCTGTTCTCCCTTCTTTA	188
	R	AACAACATAGTCCCCGAAGCC			R	GCTTTGTGGTGCTTGGCTTTT	
Kcnn1	F	TTAACCGCGTCACCTTCAACA	157	Taar2	F	GAGGCTTACGCTGATGGAATTG	147
	R	CTGGTCACTTCCTGCTTATCGT			R	GCCGTAAATCCCCACCATCAT	
Pde3b	F	CCGTCGTTGCCTTGTATTTCC	164	Taar3	F	GCGAACACAAAAGGAGCAGTAG	191
	R	CTTGGGTCAATCAGCAGGTCT			R	TACCCGAGCCATACCAGAAGAT	
Pde6d	F	GTGGTTCTTCGAGTTTGGCTT	119	Taar4	F	GGCCCTCTCAGAAAGCAAAATG	104
	R	TGATGACATTGCCCGTTAGGA			R	AGGGTAGCCAACACAACACAA	
Pde10a	F	GGACAGAGACAAGCGAGATGAA	160	Taar5	F	TGTCAAGCGGGAAAGAAAAGC	126
	R	GCGAATTACCTTCTCCCACTGA			R	AGGGGTGGGGTGATGAAGTTA	
Plcb3	F	TTAATCGGCGGCACATCACT	133	Taar6	F	CAGAGTGGCGAGAAGAGAAAGAA	137
	R	AGCTTGGGTTCCTCTTCCTCTA			R	AAATGTAGGCAGGGGTGATGAAG	
Plcb4	F	AGTGAAGGCAAGGAAGGACAAG	154	Taar9	F	CCTCCTTCTGTTTTGCGTCTCT	119
	R	CGCTGCAGACACACAATATCC			R	GCACAGTCCAGAAACCGATACA	
Plcg1	F	CGACAGCACCAAGCAAAAGAC	116	Vmn1r1	F	CTGCTCTCTCTGGGTTGTTAGT	170
	R	CAAAGCGCAGAAAGGCAAACT			R	GGGAAATGCTGGTGTTGTGAA	
Pld3	F	TCCTTCTACTGGACCCTCACAA	124	Vmn1r26	F	TTTATTCCTCCGGTCTGTGCCA	100
	R	CAGCGATGCGAACCTTTACAC			R	TAGTGGACCTGTATGGTGGAGAT	
Reep1	F	TACAAGGCTGTGAAGTCCAAGG	157	Vmn1r54	F	ACTACATCGTGCTCTCTGGCA	180
	R	GCCAGGCTACAAACGCTATTT			R	GAGGGAAAAGCTGGTGATATGG	
Reep2	F	ACCCTGTACCCAGCCTATTCTT	157	Vmn1r65	F	GAGGACAACAGAAGAAGTGGCT	142
	R	GCTCAAAGTAGAAGGGGAACCA			R	TTGGATGATCTGAATGGGCCTC	
Reep3	F	GTGGTGCTGGTGTTTGGAATG	121	Vmn1r66	F	ACATCCACAGCTCTCAGGTTTC	164
	R	CAGTGTAGAGGGCAAAGACGAT			R	GACCATCACCAGACACCAACT	
Reep5	F	GTGGCTTTGTATCTGGTGTTCG	102	Vmn1r67	F	GGTGTGTACCTTCCTGGCATT	174
	R	GGGACTCTCGATGGCTTTCAT			R	CTGAGTCTGGGCACAAAAGTAC	
Rtp1	F	TACCCTCTTTCCCCACGTTCT	180	Vmn1r70	F	AGAGTTTGCAGGGGATTTTCC	142
	R	ACACATTGTGCTTGAGGTTGGG			R	AGCACAGGACCAGAGAAGAAC	
Rtp2	F	CGAGCAGTGTTACGATGAGGAT	174	Vmn1r71	F	CCAACACATCCGTAGCACTCA	109
	R	TTCTTGGAGGCATCGGTATAGG			R	GGTGAGAGAACAAAAGGCCAGA	
Rtp4	F	TTCCTCCCCATCAAAGAGCTG	170	Vmn1r72	F	TAACTCCAAAGGGCTGATGCT	178
	R	GGGCAAATGCAGCAATAGACA			R	TGAAAACCATGAGCAGTAGGC	
Scn1b	F	ACGTCTACCGTCTCCTCTTCTT	171	Vmn1r73	F	ACTAAGAGTATCAGGTCCCAGGT	101
	R	CCATCTCTGCCACAAGCCATAT			R	ACAATGCAGCTCCCACATTTC	
Sstr3	F	GCGAACAGCCTTCATCATCTAC	100	Vmn1r77	F	ATTGGCCCCTTCTGCTTAGTCT	160
	R	CGACCGCACCTTTACCACAAT			R	AGAGTACAGCTCGCACATGATC	

Vmn1r78	F	TCTACTCTGCTTCTCTGATGGCT	160	Vmn1r212	F	GGAGTTCTGACTGGATTTTGGC	135
	R	GATAGTTTTGGTGGCTTGGTCC			R	AACCTTCTTGGTGGGATCTGA	
Vmn1r80	F	GTTACGGCCTACTCCAAATACC	139	Vmn1r224	F	ACATTGGCTCCAGAACATCTCC	141
	R	GAAAGCAGGGTAGAAACAGGTT			R	CCAGCCACCAACCAGGATTATA	
Vmn1r82	F	AACTCTGGCCAACTCCTTGTC	181	Vmn1r228	F	GGGACATGGCAGTAGGAATAGT	170
	R	GATTATGGCCGCTTGGAAACA			R	GGAGAGAAGGATCAAGGCGTTG	
Vmn1r84	F	GGTCTGTGTTTGAGCATCATGG	178	Vmn1r230	F	GCCAGGAATTTGGGAACAGGAA	124
	R	TGGAGTAGGAGAGGACAAAGGT			R	CTGTGGGCTTTCGTTTGTGTT	
Vmn1r87	F	CTCATCAGAAGAAGCCCGTAGA	175	Vmn1r231	F	TCGCATGAATCAAGAGCCACT	127
	R	GAAAGGCCCCTAGTAACACTGT			R	TAATCATCCACCAGCCAGCAC	
Vmn1r88	F	CCTACGTTTGTCTCCTGGCTTA	145	Vmn1r232	F	GGAACATGGCAATAGGAGTAGGA	183
	R	CACGGCCAACGAGAGTCATAT			R	GGGGGAAACCTTTGGAGATAATG	
Vmn1r89	F	CTTCTCCTCACTCACGATCTCT	110	Vmn1r233	F	CTGGTCTCTGGCAAATGTAGCT	139
	R	CTACTGTGGAGATGCTGGGAT			R	GGCTAGAGGCTTTGGGGAAAT	
Vmn1r179	F	ACCAATCGACACTACAGAGGC	156	Vmn1r234	F	GTGCATCAGCTCTTCCCTATACT	199
	R	ACTCCAATGCCTCACAAATGC			R	GCACACAGCACCAGGGATAAT	
Vmn1r194	F	CTGTTCGTGATCTCGTCTTCCA	138	Vmn1r235	F	GGCTTCTGCTCTGTTTGTCTTG	192
	R	CTCTGATCTCTGGGCTGAAAGT			R	GGACTCCGTGGATGATTGTGA	
Vmn1r195	F	GGCATTGCAGGCTGTAAAACT	181	Vmn1r236	F	CTGTACTATGTAAAGGAGTGCCC	139
	R	TACAAAGGAGGAGGAGAGAGGG			R	CTAAGAAATGAGGTGCTGCCA	
Vmn1r196	F	CACAGTGGTCCAAGCAGTTATC	156	Vmn1r237	F	TCCTGGGCAACTCCTTCTTAGT	160
	R	GCTGTGTCTCTGATGGAAAGGA			R	TTCAACCCAAAGGCAGACACA	
Vmn1r211	F	GCTGTAAAGTTGCTGTCTACCTG	165				
	R	GGCAGAGGAGTGAGGAAAGAAT					

Table S3. Primers for OR genes used in the biological experiments for MRPrimer.

Gene		Sequence	Size (bp)	Gene		Sequence	Size (bp)
Olfr6	F	TCTCACGAGTTGCCTTCTGTG	156	Olfr345	F	CACCCTCTGCACTATTCACAAA	174
	R	CAGTGGCTGAGAGTGGGAATAT			R	CAACCAGTCAGAGAGGTCACA	
Olfr11	F	TTCTGGTGTCCCATTTGGATCC	104	Olfr350	F	CTCTTATGGTCACATTGTGGCC	181
	R	TTGTGGGACTGTGCATGTGAT			R	CACAGAAGCAACTATGCCCTTG	
Olfr16	F	TTCCTGGGATTCTCCAGCTTTG	168	Olfr354	F	CAGCCATCCCCCATTTCTACT	114
	R	TGAGCTTGCCAACATGCTCA			R	GAACAGCTAGAAACACCAAGCC	
Olfr17	F	CAGCACTGCCATCCTCACATA	149	Olfr356	F	TACGCTGCTGATTCAGCTAGC	158
	R	TCAATGCAGCCTTCACCTCAT			R	CTTTGTGGAGGTTGCAGCAAA	
Olfr38	F	CACAAGGCTGGTCATCACATC	127	Olfr368	F	GAAATGTGGGCATGGTGCTAC	106
	R	AGCAAGGGTTTCACAGGCTAT			R	GCAGAGGAATAGAAGGCATCCA	
Olfr48	F	TGTGGGCTTGACTCAGAACATG	112	Olfr417	F	ATAGACAAGGACAGCCGCATT	172
	R	GCTGCTGATGGTGACCATGATA			R	GTCCACATGCAACATTGGTCAT	
Olfr50	F	GTTCTGCTAGTGATGGTGTCCT	200	Olfr429	F	AGCCTTGGCAGTAGTCTACTCT	106
	R	GTGATGACCACCACTGCTAAAG			R	TCTCATAAGCTGCCTCTTCAGG	
Olfr51	F	ACCTGCATGCTCTCTTGCATA	160	Olfr432	F	AGGTATTGTGAGAGCTGTGCTG	118
	R	ACCAATCCACCAATGGTGAAG			R	GACAATGAAGGCAGAGCCAAAG	
Olfr65	F	ATTGCTTCTACAGATGAGCGGG	143	Olfr450	F	ATCAGGCTTCATACCCCGATGT	121
	R	GTGATGTGGACGACATGAGGAA			R	GGATGAGCTTTTGGGCTGAAAC	
Olfr71	F	CAGCAACAGAACTGCAGTCTC	102	Olfr452	F	CACAAGGCTGGTCATCACATC	140
	R	ACCAGGTACATGACTGAGCAC			R	CCAATCGGACCACAGCTAGAAT	
Olfr74	F	TGCTAGCCCTTTCTTCCTCTGA	147	Olfr552	F	GTCAGCAATGTTGTTGGCCAT	105
	R	CACTGGCTGAACGCATCTTAAG			R	AGGCCAATCTTGATGATGAGGG	
Olfr140	F	CATCCTGCATTCAACAGCTCAG	108	Olfr553	F	CAGTACTTACCCATGCAGTGGT	103
	R	GCCAGCTCCAGTAAAGGATACA			R	GGCAGCCTTAGGCGAATTAGA	
Olfr248	F	CCATCTGCAATGCCCTCAAATA	186	Olfr556	F	CTCCATCATTGTGGGCTCAGAT	141
	R	GCCTCAGAACTGCCCTCATAT			R	AGAGCCATGACCCCAACATGA	
Olfr273	F	TGTTCATGGCATCCGCATCA	136	Olfr560	F	GTCATGGAATCCTCAGTGCTGT	151
	R	AGCTTGAGGACAGCTAAGACCT			R	TCGGTGTCAACATTACAGTCCC	
Olfr275	F	GTGGAAACTGGACCTGTGATTC	113	Olfr575	F	GGCATTGGATCCCATAAAGAGC	112
	R	ACCATGGAAGTGTCTCCACAG			R	AGCGGTGCATGGATGCTAAA	
Olfr283	F	TAAGCTCAGCCTCTGGGAGAA	118	Olfr577	F	TTTCATGCTCAAACGGTTCCC	151
	R	GTCGATCCGGAATTTGGCATG			R	GTCCACTCCCACTGTAGAAACA	
Olfr305	F	TTCATGGCTCTCCTTGGATCAG	153	Olfr592	F	CCGCTTACCCTTTTGTCAAGC	110
	R	TAATCCAGTGCCCCAGGAAAC			R	AAAGGCCATACACAGCGTTGA	
Olfr307	F	CCCTTGACCTACAGCTCCAAA	145	Olfr599	F	AGTACTCGCAGCTACTGACCT	155
	R	GCACAAGCCAGAACGGAAATAG			R	GCAGAACGCCAGATTCCATTC	

Olfr606	F	GGGTAACAGGGAACTGTTCCAT	124	Olfr1043	F	GCCAAATGCGCTGGTGAATTT	181
	R	ACCATAGCAGGCAAGGACATG			R	CCGCGGCATGAGAATGACATA	
Olfr622	F	AGGTCTAGTTGGCCTGATGAGA	179	Olfr1052	F	GCCAAGCTCCCAGTATTCTGT	136
	R	GGGCCACTGAAATTCCATATGC			R	CTCTATGGCTCTCTTTGCAGCT	
Olfr631	F	CTCATTGCGCTGTCCTATGGA	137	Olfr1055	F	TTGAGACTGGTCAAGTCGCATC	107
	R	AGTCCCATCATGGGCACAAAG			R	TCTATTCAAGGCCTGCTTCACC	
Olfr648	F	GCTGGCAATGACTGATCTCATC	100	Olfr1089	F	ATAGGCATCACTAATCGGCCTG	125
	R	GAGGCAGCCTGGAAATGTGATT			R	TGCAGTCGAGGGTCCACTATT	
Olfr661	F	CTGGGCATTCTTGCTATTGTGG	145	Olfr1090	F	ACCTCAGTCCAGTCATTCTGATG	135
	R	CCATGGCAACAAAGCAATGGA			R	CTCCAAGTCCTATGTAGGGCAAA	
Olfr745	F	CGGATCCAAGAACAGCTCCAT	163	Olfr1093	F	TGGAGTTGTGCATGGTGCTAT	167
	R	CTGATCCCATCTCACAGCACAA			R	CAATCAAGCCCACCAAGTACAAG	
Olfr768	F	GAGCGTAATTGGTAACCTGACC	150	Olfr1106	F	CACTACTTACCACAGAGGGCTT	137
	R	GACGCCATTGTGTAGAGGAATC			R	TCCTAAGCACGATCCCAACAC	
Olfr788	F	CATGAACAGGAAACTCTGCACC	107	Olfr1111	F	TTGCAGATGCTGAGTGCCTTA	147
	R	AAGCACAGTAATCCTGCTGGAG			R	AGGTCACGCTTCCGCTAAAA	
Olfr812	F	ACCAGTCAGGAGAGTTAGAGTTC	128	Olfr1112	F	CCTAGCTTGTGGGGACACTTT	135
	R	GAGGATGATCGTTAAGTTCCCC			R	GCAGATGGAAGCTTCAGAATGC	
Olfr827	F	AGCCTTTATACTAGCAGCCATGG	100	Olfr1124	F	TGCTGCATTCTTATCCTGGGAG	152
	R	CAACTGGACACAGACACTCCTT			R	CCACTTATCCAGGAGCCAATCA	
Olfr855	F	GACTGACAAGAAACATGGAGCC	109	Olfr1128	F	GGGGTCTTTGGAGATACAGAATG	138
	R	AGGAAGAGGCTGAAGATGAGAC			R	TGCATAGGCAACAGCCACAAG	
Olfr885	F	GGTGCCTTGTCCCACATAGTT	168	Olfr1133	F	CCTGTGCATTCAGATTGTCCTTC	145
	R	GGGCACTGTTATATTGACACCA			R	CACCCTACCACACAGCCAATTA	
Olfr933	F	GACTTTGCTCACTGCTAGTGT	167	Olfr1136	F	GTTGGCTGTTTTCTCCAACTCC	136
	R	CAGCTTTATGTCAGAGCAGGAG			R	CTCTACTAGACATGTCCACAGCA	
Olfr988	F	CATGGGCTTCCTAAATGCTTCTG	184	Olfr1152	F	ATGGACCATCAGCTTCACATCC	116
	R	TACAGTGCTCACTAGGTTGAACC			R	CTCTTGGCAAGCAGGTCCAATA	
Olfr992	F	TCACAGGATGCTTGGCTCAAT	159	Olfr1157	F	CCCTGGCATGTATGGTCCAAT	168
	R	CAAGCAGCAGCAAAATGCAGAG			R	GGTAGCATCCAGACACCAGTAT	
Olfr1009	F	TGATCGGACCCTATGCTGTTG	191	Olfr1161	F	CTCAAGATGCAATCATCCAGGGG	144
	R	AATCAATACAGCGCCTGCCA			R	CACTTTGAATGTGAGCTGGGAG	
Olfr1010	F	GCGGTGTTACAGCAAGTACTCT	107	Olfr1162	F	GCAAGTCGTTAAAGTGGCCTC	134
	R	TGGAGTCGTGAATCAGCACAG			R	ACTGAAGAGGGATCTTTGTGCTC	
Olfr1014	F	CTGGATCTTGGATTGTCCACAG	129	Olfr1164	F	GGGCTGGGATATTTTCCTCAAC	149
	R	CTCAGTATACCCAAGTCCAGCA			R	CAGGCATGCCATTTGACTGAA	
Olfr1022	F	CAACACTGCAGTGATGGATTTC	146	Olfr1176	F	TCCCCACTAAAGGAGCACTACA	120
	R	GGGAATTGGTCCTGATCAGCA			R	GTGAGCTCTTTGACTTGAGCAC	
Olfr1030	F	CATATGCTGCGTGTTTAGTCCAG	193	Olfr1179	F	CTTGCTGTGCTGGTGCTTTTA	185
	R	AGGCCACTAAGGAAGCCATAGA			R	CAGTCCCATCATGCCTGAATTG	

Olfr1180	F	ACATGCACTCAGCTCATGGAC	104	Olfr1278	F	ATTCCAGGGTTGTGTCTTCCAG	170
	R	TCAGTCAGGAGTTTGGGAGTCA			R	CCCAAGCACCAGATAGAAGCATA	
Olfr1184	F	CATGCTACAGGTCTTTGCCATG	144	Olfr1279	F	GAACGATGTGTGGGTTTCCTC	131
	R	ATCAGGATATGGCACCTGCTTC			R	TCTCCACTCCTCCCATAATGTG	
Olfr1208	F	GGGTGATGTCCATTCTGACCTT	195	Olfr1289	F	TAACCCTTGTAGCCATGGCCTT	116
	R	TGTCGTCCCCAACAGAATCAC			R	AATTGCCCATGCAGTAGCCA	
Olfr1230	F	AGGCCTGTGGTCACTTATCCT	129	Olfr1301	F	GACCATTGTGTTGGTGCAGTA	183
	R	CCACAGTTTCCTCAGAGCATGT			R	CACAACTGCCAAAAGCTGACT	
Olfr1234	F	GCAAGGCCTACATCCACATTTC	164	Olfr1305	F	CCACGGATGTGCCTGCTAATA	158
	R	AGCCCACTTACGATGGAGCTA			R	GTGCATGCAAGCTTGACAAGT	
Olfr1239	F	GTTGTGGTCAGCCCAAGTTTAG	171	Olfr1311	F	ATGGGGAATCCAGTTGCTTCTC	110
	R	GTGTCCTATGAAAAGCTGGCTC			R	AATGCAGGTTGGAGTCAGCAG	
Olfr1240	F	AGTCATCGTTGTTTGCTACCTC	101	Olfr1340	F	GACCTGGATTTTTGCAGCTATGG	101
	R	AAGTGTCAGTACATGCTAAGCCC			R	CCAGGGCTATGCATAGGGTTT	
Olfr1258	F	GTTGAACTTCCTCCTTCTGTTGG	174	Olfr1389	F	AAGGCTGGATGTCAGATTGCA	178
	R	GACAAAGTGGCTGAAGGTCTCA			R	GACCAAGGCAAGGAAAATGAGG	
Olfr1260	F	ATGTGGCTTCCTGGTTTCCAC	133	Olfr1404	F	ATGGCTGCACTGCAATCATCT	125
	R	GAGCGGGAAAAGGTCACAGAT			R	CCGCAGAGTGTATACCAAAGGA	
Olfr1262	F	GCACCATATCCTTCAATGGCTG	183	Olfr1406	F	GCCATATTCAGGCTGCCATTT	141
	R	CGATTCCAGCTGCTACCACTAA			R	GGCTGTGATGACCATAAGGCTA	

Table S4. Primers used in the comparative biological analysis.

		MRPrimer			PrimerBank		
No.	Gene	Sequence		Size (bp)	Sequence		Size (bp)
1	Olfr5	F	GCTAGGTGGGCTATTGGTTTCT	186	F	TTGTGGGCACAGAGTGCATT	133
		R	AAGCAACCCCAAAGGATGACA		R	CCTAGCCATGAAATCATAGCCAA	
2	Olfr39	F	CATGCAGTGCCTCACTCAAGT	166	F	CAGATGATCCTAAATTGCAGCCT	113
		R	CCAGCACATTAGCACAAGGAAG		R	GAGATGGGAATCAGAACTGACAG	
3	Olfr49	F	ATTCCTGGGCTTTCTCCTGAC	185	F	CTTCCCCAAGATGCTAACCAAC	172
		R	GTCCTGTGATGATGTTGGTTAGC		R	GGTGGCATAACGCAAAGGG	
4	Olfr78	F	CATGCCACCTTCCTGCTTATTG	138	F	ATGAGTTCCTGCAACTTCACC	111
		R	GCTCCGCTCTGTTCTCACTAT		R	TGCTACAGCATACATGGAAAGC	
5	Olfr97	F	TTGGGAATCAGTGCACTGCTC	163	F	AGCCTTGGCTACATGCAGTTC	158
		R	GGTAGGTAGCAAAGACTGTGCT		R	TGAAGGGGTTTAGAGTGGGAG	
6	Olfr118	F	TGCTCCCCACTCCATTACTCA	233	F	CACTTGCTTGTGGCGATACAT	153
		R	ACTACGGCCACAAAGATTGCA		R	CTTTATGGCGACCCTCAGGTG	
7	Olfr122	F	GCTGGTAATGCCCTCACGT	109	F	AGGAGAACAGCTTGTCTGTCA	60
		R	CCTCAAATAGGTGGCAGACGT		R	CTCCAGGGACCTCAGAGAACT	
8	Olfr130	F	GTCGCACATGCTGGTAGTAGT	223	F	TGACACTGGTAGGCAACACAG	176
		R	AAGAATTTTCTTCCCAGTGCTGT		R	TGTGGCAGTAATTGTCTTGGC	
9	Olfr190	F	CTGGCTTTTGTGGATGCTTCC	162	F	CTGTGACATCGTTCCATTGCT	263
		R	TGCCATAGCTCCCAACAAGAA		R	CTTGGGACACGGGGAAAATATAC	
10	Olfr218	F	GAGCATGGCTATTGTCCAGGTTA	168	F	CTGCAATCCTCTAAGGTATTCGG	102
		R	AACACAAGGACACACACGCT		R	ACCTGGACAATAGCCATGCTC	
11	Olfr222	F	GGTCATCCTGACAGTGCAATT	114	F	CACCCCAAAGGACTTTATCCTC	103
		R	GCATCATTCGCCGTAGTATCAG		R	CCCCAGCATAGCCAGAATGT	
12	Olfr235	F	GGCTGTAACCTGGAACTTTTCC	127	F	GTGTTTTGGAATAGCAAATGCCT	180
		R	GGGGGCTGTGGAAGTGATATAG		R	GAGCTGGATCGCAAATAGACAA	
13	Olfr267	F	CTGACTGCTCTGCTGGAAACTA	168	F	ACCCGAGGTTAGAGATTGTTCT	111
		R	TGGAATGGGCAGAAGGAGAATAC		R	TTTGAAGGCGTGAATCCAGGA	
14	Olfr340	F	CATCAGTCGCATCTCCAAAAATG	250	F	TTGGGACTCCCCATTCGAG	195
		R	AGGGAGTTGGCAGTAGATAAAGT		R	TGGAGATGCGACTGATGAGAA	
15	Olfr348	F	GTAACACTGTCCACCACTTCTTC	161	F	ACCCCCTCCACTATACAAGAATC	89
		R	AGTGGCTCCAATGCGTCCATA		R	AAGGGCACCAGCAAATGATAA	
16	Olfr355	F	TCCTTTCCCCACTTCCATTCAC	198	F	GATGTGCCCTACTGGTGACC	336
		R	AGCAATGCATAGGAAGGGAGTC		R	AAAAGAGGGTTACCACAGTGAAG	
17	Olfr362	F	TGCCCCACACTTTCCTCTTTT	136	F	TGTGCCCCGAATGCTTCAC	122
		R	GAGAGGCCACGCAAGAGATAAT		R	TTCCCCAATATGGTGGTCAGATA	

18	Olfr401	F	CCCACAGGCTTACAGTTCCAT	187	F	TATTCCCTCTATGTTGGGTCGG	150
		R	CAGGATGAGGCCACCAAAATTC		R	CAGATGGCTAGGAAGCGGT	
19	Olfr424	F	GGTTCCTGCATCTTTGGCTTTC	124	F	GGATGGATACCCGTCTTCACA	127
		R	CCAAACGCAACACAGGTTCAA		R	ATGGTCCTTTGCTTACTGATGAG	
20	Olfr433	F	GGGCACTGACAAACTTATTGCCT	119	F	CATGTCTCACTGCCAGTTTACC	105
		R	TCTTTTCACTGACCCTCTTCACG		R	CATGCCACCTTGTGCTCAC	
21	Olfr453	F	TCTGGGCTTAGGAGGGATTGA	145	F	CCTGGTGGATGTGTCTTATGC	135
		R	ACCACCGACCCAAGAAACAAT		R	AACTCAATCCCTCCTAAGCCC	
22	Olfr460	F	GTGGGAAACACGGTCATCATTG	127	F	TCCTGGCTCTGTAAACCTACG	188
		R	GCATCACGGGCACAATAACAG		R	CGGGCACAATAACAGTTGTAACC	
23	Olfr461	F	CCTTTTTGCGACCTTCCTTCT	181	F	GGCACCAAAGAACTACACCAC	109
		R	AAGAAGCCCCTGGAGCATATA		R	GACGTTTGTCAGCATGGACTA	
24	Olfr510	F	TGGCTTCCATTGACATAGCCA	100	F	ACCACACTGTAGTCACAGAGT	123
		R	ATGCCACACCCAATGTAGGAT		R	TGGTGCTTAGATTCCCAGACA	
25	Olfr539	F	TCTGTACCTCTTCTGTGATTCCT	245	F	GTGGTCCCAAGGTTATCACCC	156
		R	AAGACCGATGAACCATACAACCA		R	TGCAGCCATAAGACAACAAGG	
26	Olfr558	F	TCCCTTTGTGTTCCCTCTACCT	153	F	TCAATAGCAATGAATCCAGTGCC	114
		R	TTGGCATGGATGAGGTGGAAA		R	GCACAGCAATAAGGTAGAGGGAA	
27	Olfr569	F	GCCTTGTTGGCTATCACTGAC	109	F	GGAATCCCAGGGTTGGAGAAT	87
		R	GGATGAGGCAGGCGTTGTATT		R	GGTGATATTTCCAGTCAGTGCC	
28	Olfr611	F	GAGGAGGCTCTACTTTTGTCGT	134	F	TTCCCCACACTGTTGAGAATCT	141
		R	CCAAGGTAGAGAGCACCACAA		R	CACATACCAATCGAAGGCCAT	
29	Olfr613	F	TGGTTAGAGCGGAGCAGAATC	247	F	CCTTCTGGTTAGAGCGGAGC	110
		R	AGTGGAGCACAGATAGCAACC		R	CCAAGACACTAGGCATTGTTGAC	
30	Olfr619	F	GCTCGCATACTTTGTGCTGTG	215	F	GGCTCACCTATTGTGGGAAGA	74
		R	GGATTGAGGGAGGGTGGTAGA		R	ACAGGCCAACCTGGCAATG	
31	Olfr632	F	CTTCTGTGCTGGGGGTGTTAT	128	F	ATGAAGGTGTCTATTCCACCACG	100
		R	GTCCAGAGCCATAGCAAATAGC		R	GCAGTGAAATCCAATGATGAGCC	
32	Olfr635	F	AAACACCACCATCCTAACCGTTA	188	F	CCATCCTAACCGTTATCCGCA	117
		R	GGAAGAAGAACTGGGCAAAACA		R	GCATGACTGTAGGGAGTGTGG	
33	Olfr653	F	CTTCCACCCTCCCACATTTGT	112	F	CCACCCTCCCACATTTGTTTT	78
		R	ACCATTTCCAACCAGAGCAAGG		R	GAGCAGAAGGGAATAGCAATCC	
34	Olfr677	F	AGCTTGTGCACCCATCAAGAT	156	F	CCTTGTGGGAAACATCACCAT	175
		R	GTGTTTAGCGCCTTCAATCGG		R	GCCCCCGAAACTGATCTCC	
35	Olfr693	F	CGCCTCAGTTATCAGTCCCAAA	107	F	CCATGTACCTCTTGCTTGAGC	67
		R	TACCCAGTGCCAGTTCCAAGAA		R	TTGGGACTGATAACTGAGGCG	
36	Olfr694	F	CAGCCCTGTACTTTTTAGCCATA	122	F	GAGCTGCTCTGTGCCACTATC	173
		R	AAGCAAGTCCATGAGAGAGAGC		R	ATGGCCTTTGGAGTGATGACT	
37	Olfr740	F	TCCTGTTCGTTCCTTTCCTCTTC	178	F	CCCGACCTCTGAGCATGAAG	83
		R	TCAGAGGTCGGGCTTAGATACA		R	TCACAGGATTAACGAGTGGAGT	

38	Olfr799	F	TCCATTGCCTATGCTGCTTGTA	214	F	ACAGATGACATTAGGCTGCAAA	227
		R	GGCTAATTGGTGGGAGAACGA		R	GCAATGGACTTATCCCCAGATG	
39	Olfr828	F	GCTCTTCATTCACTACCTCAGTC	144	F	GGACTGATGGTGTTGCGGTT	444
		R	TTGCCCTATTGATGTGCTTCC		R	CCTATTGATGTGCTTCCTCAAGG	
40	Olfr830	F	TGTTGGCCATCAAGTGTGACTT	182	F	CCTTATGAACCCCAGTTTCTGTG	75
		R	AACTACAACAAAGCAGGCCTGG		R	TGCAGCAGACCATTTACAATACT	
41	Olfr911-ps1	F	TGGGGCTGGAAAATGGTTCTT	124	F	TTGTGCCCAGTGTTATCATCTTT	120
		R	TTCCCCACTGCTGTTGTTGT		R	CAGCAAGTATATGGGAGCTACAG	
42	Olfr918	F	CCACAGGTCTGCTCAATGCTA	154	F	TGCTACTCTTCTGTATCCAGTCC	251
		R	AAGAAAGTTGGAGGAGGGGCA		R	CCAGCAAATGCCATCCCATAAG	
43	Olfr960	F	AGCAACCTCTCTATCTCTGACAT	148	F	AGCCTGTGAAGATTCCTCTCT	230
		R	AGCACAAAACGCATCCAATACA		R	GGTTGGGCTGTAGGTAGATGAC	
44	Olfr967	F	GATGTCCTATCAAGTCTGCACC	175	F	GCTGGATTAACAAGCACACCA	72
		R	AAAAGTAGGGGAGCAAGAAAGC		R	CGTTACTGCATAGATTCCGAGG	
45	Olfr978	F	TGGCCCTGGTCTCTTCATCTA	174	F	GGCCCTGGTCTCTTCATCTAC	63
		R	TGAGGAAGCACAGACCCATAT		R	GGCCACAATTCCATCTACAGC	
46	Olfr995	F	GTTGTCCCAATCATCAGCCTTTC	100	F	TATGCCACATTTGCGACCAGT	326
		R	TGACCAACACAGTGAACGTCA		R	AACACAGTGAACGTCAGGTTAAA	
47	Olfr1033	F	ACCTTACAGCAGTTGGCATATTT	195	F	TCCACCCCTCATCAAGATGG	258
		R	ACTTCTTGGCAATCACTTTGTCC		R	GACTCCTCAGTGGGTCGTCT	
48	Olfr1053	F	GTGAATGTGCTACCCAGTTGTC	172	F	CATCACTGCCTGGGTTCATCT	108
		R	CTGTAGAGGTACGGGATGCC		R	GGTTGCCCATGACTGTGACT	
49	Olfr1079	F	TCCTGATCATCCTTGGCTCCTAT	128	F	ATGTCACAAACTGTATGTTGGGT	253
		R	CCCATAAAAGACAGAAACCACGG		R	GCAGATAGGAGCCAAGGATGA	
50	Olfr1145	F	TGACTCAGACTCCGACTAAATCC	120	F	CTGTCTGCACTGTTCTCCATC	391
		R	GGAGATTGGGAAGAGCAGAGAA		R	TCTGAATTGGAATGCCACTTAGC	
51	Olfr1204	F	CCGCAGTTGCAGAAAATCTTG	201	F	TCACACAGAATCCGCAGTTG	113
		R	ATGGAAAGTATCAGCAAGCAGTT		R	CAGTTGGCTGTTTGTAATGGTG	
52	Olfr1225	F	CCACAGCTCTGAAGGGAAATTT	171	F	TGTGGCAATCTTGTGATGGTG	265
		R	GAATTGAGCAATGGGGTCAACAC		R	TGTGGGCTTTCAGAGAGTACA	
53	Olfr1255	F	CTCTTGGTCTCTTTGTTGCTGC	229	F	AAAAGGAACGTGACTGAGTTCAT	244
		R	GCTTTATCAATTGGCAGAGTGGT		R	GAAGGGAGTCCGCAATCAGC	
54	Olfr1284	F	GTAAGCATCATCGTGGGAAACC	133	F	GGACTCTCCAGTTCTTGGAAAAA	78
		R	TGGGCACTGTGGTAGAGGAAAG		R	TCCCACGATGATGCTTACATAGA	
55	Olfr1295	F	CTCCAGGGACTTTCCCACTCA	198	F	GGGGTTGTGGTTGTAACTTGC	104
		R	AGGAGTGATGTTTGAGGAAAGAC		R	GACAAAGCCTTAGATGCTCCAG	
56	Olfr1297	F	CATTGCTTCAGGAGAGGTGGTAT	178	F	GGTATTGTTGGCTTTAATGGCCT	372
		R	CTGCAAAAGCACTACCACGTG		R	GCCTTGGAAGCTCCAGTTTTC	
57	Olfr1303	F	CTGCTCTGTTACTTCCCCCAA	120	F	GTGTCTGCGTTTGTGTTTCTG	106
		R	ACCATCTCCACTCCACCAACT		R	GGATGTTTCCAGCCATGCTTAAT	

58	Olfr1331	F	CCTTTGTTACCACCACCATGC	114	F	TTTGGTCGTTACTCCAATCTCCC	240
		R	TTATACCCAGGCCACCGAACAT		R	AGCAATGGTGTAAAAGCACTGT	
59	Olfr1336	F	CCTCTTCTTAACTTGTCCTGCAC	142	F	GAGTTGGGCAATGTGACCAGA	249
		R	ATGGCATGCCTAGAACTGTCCT		R	CCCCATGAGTAGTGTGGGC	
60	Olfr1344	F	GCTTTGCACTCTCTGTACCCA	200	F	GAACCCTCAATGATTCAGGAACC	129
		R	AGAGGCTAGGATGAGGACCAAA		R	GGGCACCATTACCCAGCAC	
61	Olfr1353	F	TCTGGTTCTGGTGTCTTGGATTG	101	F	AACTTCTTGCTCACTATCATGGC	584
		R	AGTGTGGGATTTCTGGCTGTG		R	ACGAAGAGTCTTTTTAGGGCAC	
62	Olfr1356	F	CAGTCCTTCTTCTTTGGGTTGC	145	F	AACCTGTCCATAGCTGACATCG	76
		R	CGATGTCAGCTATGGACAGGT		R	TGCTTTGTGTGCGGATATTCT	
63	Olfr1358	F	GCCTTGATTGAGACCTGCATGA	144	F	CCATCCCTTGCGCTACTCTG	112
		R	GCTCAGGAAGAAGATGCCAAGT		R	GAAGATCATGCAGGTCTCAATCA	
64	Olfr1388	F	GGCCTTCTTTTTGGTGGGATTC	127	F	TCTCTCTCGACTGGACCTTCG	153
		R	GTCCAGTCGAGAGAGAGCAAT		R	ACACACCTTTCATAGCTGATGG	
65	Olfr1392	F	CTAACTCTCTTTGGGAACACTGC	203	F	CTGGCCTGCACTAGAACTCAT	78
		R	GTTATGAAGAGCTGAGACACACA		R	ATGGCAGTGTTCCCAAAGAGA	
66	Olfr1408	F	ACAGTAAGGACCAAGACCAGCT	125	F	AAGATTGCCTCATCTGATGGC	243
		R	CCTACACAACACTTTCCGCAGA		R	CCTACACAACACTTTCCGCAG	
67	Olfr1411	F	CTCTCATATGATGGCTGTGTCCT	102	F	CTGCCATTGCCCCTAATGC	210
		R	ACAGATATCACCTTGCCCTGT		R	CCAGACGGGTACAGGTTGTAG	
68	Olfr1420	F	ATTAACCACTCACTCCACACCC	185	F	GGGGTGCAGATGGTGATTTTT	177
		R	TCAGCTCCACCCAAGAAAACA		R	CAGTGGTGCAATGGATGATGTAT	
69	Olfr1443	F	GACTGGAAACTTGGGGATGCT	121	F	ATGGAGAACAGGACAGAGGTG	143
		R	TGGGGTAACAGCAGAGGAGTA		R	AGAATCAGCACAAGCATCCCC	
70	Olfr1444	F	TTTCTTCTTCGTGGGGTTTGC	154	F	GGTTGACAGATGACCCCAATC	117
		R	GCCACAGGTGTAAGAGCCAAT		R	GGTGGGAATCCGAGAAGATGA	
71	Olfr1465	F	CATGAGTGGGCTCCTAAAAGGA	189	F	ACATTGGGGACACCTTCAATC	254
		R	AGACACACACACACACCTGAA		R	TGCGGCACAAGTGGATACAG	
72	Olfr1494	F	AGTCCCATTCCTGCTGATTTGT	167	F	CCCTCTACACTACAGCCTCAT	147
		R	AGCGGGGCCTCAGATATACA		R	TGGTTGATTTCCTGGTCATGTC	
73	Olfr1509	F	CAACTGGGTGTTGGAGATTCTG	109	F	CAACTGGGTGTTGGAGATTCTG	108
		R	GAGACGTGGCGTGAAGACTAT		R	AGACGTGGCGTGAAGACTATG	
74	Olfr1511	F	CGCCAGCAAGGTTATCGCATT	165	F	TTCTGGGTGTGCTCTCCTTC	101
		R	GTGCACAACTTCCCATTCATGA		R	ACCAAATGCGATAACCTTGCTG	

Evaluating the computational efficiency and scalability of MRPrimer

To demonstrate the computational efficiency and scalability of MRPrimer, we measured the elapsed time required for design of complete sets of validated primer pairs for the human and mouse CCDS databases. We conducted most of the computational experiments on a MapReduce cluster of one master node and 40 slave nodes, in which each node consisted of two Intel Xeon 8-core 2.6 GHz CPUs with 64 GB memory and a 6 TB HDD. Those nodes are connected with each other via a 1 Gbps network. All computing nodes were running on CentOS Linux version 6.4 and Apache Hadoop version 1.2.1. For the Hadoop configuration parameters, we set the number of Map per node to 4, the number of Reduce per node to 4, the Java heap memory size for Map to 8 GB, and the Java heap memory size for Reduce to 16 GB.

MRPrimer exhibited good performance in terms of computation time. Table S5 shows the elapsed times of MRPrimer at each step for human and mouse CCDS data sets. Even though MRPrimer designs all feasible and valid primer pairs, without omitting any, it finished within 1 or 2 hours. Once the results are obtained, users do not need to run the analysis again, unless the filtering constraints are changed; instead, they simply pick the appropriate primer pairs (usually the top-1 primer pair) for use in their experiments.

Table S5. Elapsed times (sec) of MRPrimer from Step 1 to Step 7 for human and mouse CCDS data sets (Steps 4 and 5 are performed twice at #mismatch = 1 and at #mismatch = 2).

	Step 1	Step 2	Step 3	Step 4	Step 5	Step 6	Step 7	Total
human	69	60	65	3648	56	586	29	4513
mouse	50	50	59	2590	55	224	30	3058

MRPrimer also exhibited a fairly scalable performance in terms of database size (i.e., the number of DNA sequences). To demonstrate this feature, we used a much larger DNA database: 105,180 DNA sequences of *Homo sapiens* from the Ensembl site (http://asia.ensembl.org/biomart/martview/). Figure S4 shows the elapsed times of MRPrimer as the number of sequences was varied from 12,500 to 105,180 (i.e., an entire database). Even for 105,180 sequences, MRPrimer designed all feasible and valid primer pairs within a reasonable time of less than 7 hours. Because MRPrimer is based on MapReduce, users can reduce time easily by simply adding more computers to the cluster.

Furthermore, MRPrimer was very efficient in terms of computational resources, i.e., the number of computers or the computing power of each computer. To illustrate this feature, we performed the same experiments for human and mouse CCDS data sets with a small-scale cluster of commodity PCs. The cluster consisted of one master PC and ten slave PCs, each of which had an i7-4770 4-core 3.4 GHz CPU, 16 GB memory, and 3 TB HDD. Here, we used the same number of mappers and reducers (four and four, respectively), but smaller Java heap memory sizes (4 GB for Map and 8 GB for Reduce) due to the small memory capacity of the PCs. Table S6 shows the elapsed times of MRPrimer, which still finished within 2 or 3 hours.

Table S6. The elapsed times (sec) of MRPrimer from Step 1 to Step 7 for human and mouse CCDS data sets with a small cluster of less powerful computers (Step 4 and 5 were performed two times at #mismatch = 1 and at #mismatch = 2).

	Step 1	Step 2	Step 3	Step 4	Step 5	Step 6	Step 7	Total
human	303	106	165	7583	56	557	69	8839
mouse	259	91	110	3880	50	272	70	4732

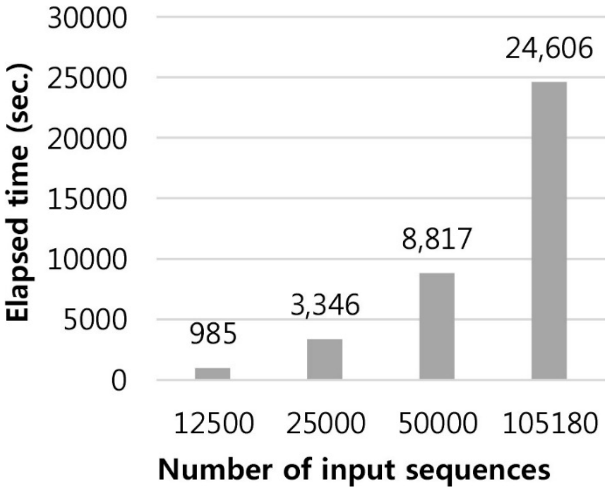

Figure S4. Elapsed times of MRPrimer as varying the database size.
